# Abundance, chemical structure, and light absorption properties of humic-like substances (HULIS) and other organic fractions of forest aerosols in Hokkaido

**DOI:** 10.1038/s41598-022-18201-z

**Published:** 2022-08-23

**Authors:** Sonia Afsana, Ruichen Zhou, Yuzo Miyazaki, Eri Tachibana, Dhananjay Kumar Deshmukh, Kimitaka Kawamura, Michihiro Mochida

**Affiliations:** 1 Graduate School of Environmental Studies, Nagoya University, Nagoya, 464-8601 Japan; 2 Institute of Low Temperature Science, Hokkaido University, Sapporo, 060-0819 Japan; 3 Chubu Institute for Advanced Studies, Chubu University, Kasugai, 487-8501 Japan; 4 Institute for Space-Earth Environmental Research, Nagoya University, Nagoya, 464-8601 Japan; 5 Present Address: Institute for Space-Earth Environmental Research, Nagoya University, Nagoya, 464-8601 Japan; 6 Present Address: Space Physics Laboratory, Vikram Sarabhai Space Centre, Thiruvananthapuram, 695022 India

**Keywords:** Atmospheric chemistry, Atmospheric chemistry, Atmospheric chemistry

## Abstract

Atmospheric organic aerosol (OA) are considered as a significant contributor to the light absorption of OA, but its relationship with abundance, composition and sources are not understood well. In this study, the abundance, chemical structural characteristics, and light absorption property of HULIS and other low-to-high polar organics in PM_0.95_ collected in Tomakomai Experimental Forest (TOEF) were investigated with consideration of their possible sources. HULIS were the most abundant (51%), and correlation analysis revealed that biogenic secondary organic aerosols significantly contribute to HULIS. The mass spectra obtained using a high-resolution aerosol mass spectrometer (HR-AMS) showed that HULIS and highly polar water-soluble organic matter (HP-WSOM) were substantially oxygenated organic aerosol fractions, whereas water-insoluble organic matter (WISOM) had a low O/C ratio and more hydrocarbon-like structures. The WISOM fraction was the predominant light-absorbing organics. HULIS and WISOM showed a noticeable seasonal change in mass absorption efficiency (MAE_365_), which was highest in winter. Further, HULIS were shown to be less absorbing than those reported for urban sites. The findings in this study provide insights into the contribution of biogenic secondary OA on aerosol property and radiative forcing under varying contributions from other types of OA.

## Introduction

Organic aerosol (OA) is a predominant part of atmospheric aerosol. The direct and indirect effects of OA on Earth’s radiative forcing have a substantial role in regional and global climate systems^1^. The effects on radiative forcing occur in part through the absorption of solar radiation by organic carbon. This light absorbing OA is known as brown carbon (BrC). These compounds absorb light in near-UV and visible spectral regions, which also influences atmospheric photochemical processes and leads to the formation of secondary organic aerosol (SOA)^2,3^. The light absorption property of OA depends on its complex chemical composition, which also depends on their sources. The relationship between the light absorption of OA with their chemical composition, sources and abundance are not well understood to date^4,5^.

Analysis of the composition and properties of OA was performed for fractions collected from OA according to their physicochemical characteristics such as volatility, solubility, and polarity. Atmospheric humic-like substances (HULIS) is a less polar fraction of water-soluble organic matter (WSOM) and recognized as a substantially large fraction. HULIS are ubiquitous in diverse environments (e.g., fog, clouds, rainwater, snowpacks, and atmospheric aerosols) and are of great interest because of their role as surfactants, their light-absorbing capability, and their adverse health effects^6,7^. HULIS are operationally defined and determined by different isolation procedures such as ion exchange chromatography (IEC), reversed-phase liquid chromatography (RPLC), size-exclusion chromatography (SEC), and solid-phase extraction (SPE)^7^. The organic fractions collected as HULIS depend on isolation methods^7^. Among these methods, SPE using Oasis HLB columns has been widely used, and the average concentrations of the HULIS from this method ranged from less than 0.1 μg m^−3^ to more than 10 μg m^−3^ at different remote/background sites and polluted urban areas and contributed to 19–72% of WSOM^7–9^. It has also been reported that the concentration of HULIS show seasonality^9,10^. Various analytical methods including aerosol mass spectrometry (AMS), electrospray ionization (ESI) coupled with an ultrahigh resolution mass spectrometry (UHRMS), Fourier transform ion cyclotron resonance mass spectrometry (FT-ICR MS), nuclear magnetic resonance spectrometry (NMR), and Fourier transform infrared spectrometry (FT-IR) have been applied to quantify and characterize HULIS^11–14^. Reemtsma et al.^15^ reported that HULIS have molecular formula similar to aquatic humic substances but with lower molecular weights. Chen et al.^11^ found in Nagoya, an urban site in Japan, that HULIS with neutral nature have low O/C ratios (mean: 0.4) and are abundant in aliphatic structures and hydroxyl groups, while HULIS with acidic nature have moderate O/C ratios (mean: 0.7) and contain relatively large amounts of low-molecular-weight carboxylic acids and alcohols. Zhang et al.^16^ found that HULIS from biomass burning sources mainly have O-containing, aliphatic C–H, and aromatic C=C functional groups. Sun et al.^10^ identified species of HULIS in less polluted (~ 1800) and highly polluted (~ 2800) samples. Despite such former studies, the abundance and chemical structural characteristics of HULIS remain largely unknown.

In addition to HULIS, OA are also composed of highly polar water-soluble organic matter (HP-WSOM) and water-insoluble organic matter (WISOM). Zhou et al.^17^ found that the HP-WSOM fraction was most abundant in Beijing, whereas Chen et al.^11^ found that WISOM was most abundant in Nagoya. Both of these studies reported that HP-WSOM has high O/C ratios (mean: 1.37 and 0.99), while WISOM has low O/C ratios (mean: 0.17 and 0.14) ^11,17^. Only a few studies have been reported about the abundance and chemical characteristics of these fractions, although the information is essential to characterize total OA in terms of the fractional contributions of HULIS and non-HULIS fractions.

WSOM is considered as a strong contributor to the light absorption of aerosol. The amount of solar radiation absorbed by WSOM relative to that of black or elemental carbon (BC or EC) were up to 40% over the whole solar spectrum and varies largely depending on the source and composition of WSOM^18–22^. As a major component of WSOM, HULIS is also considered as a significant contributor to the light absorption. The light absorption of water-insoluble OA fractions, on the other hand, has not been studies widely. Chen et al.^23^ and Huang et al.^24^ extracted and fractionated OA into different fractions sequentially, measured the light absorption for each fraction, and reported that WISOM contributed to the total light absorption more strongly than HULIS or HP-WSOM. Chen et al.^23^ showed that in Nagoya city, WISOM had higher contribution to the total light absorption of OA than that of WSOM and was dominant in the visible region. Hence, it is important to quantify the contribution of all extractable OA to the total light absorption by OA. Knowledge of the abundance, composition, and light absorption of all the OA fractions are crucial to understanding the contribution of OA to atmospheric aerosol and Earth’s radiative balance.

In this study, the atmospheric concentrations, chemical structural characteristics, and light-absorption properties of HULIS and other parts of OA with low and high polarity in PM_0.95_ (particles with a diameter smaller than 0.95 μm) at Tomakomai Experimental Forest (TOEF) were investigated with consideration of the possible sources of the OA fractions. The seasonal variations of OA, and the contribution of OA fractions to the absorption of solar radiation over TOEF were characterized. TOEF is a cool-temperate forest in Hokkaido, northern Japan. A previous study found that atmospheric aerosols at this site are influenced by local natural sources and biogenic secondary organic aerosol (BSOA) that affects the optical properties of aerosols^25,26^. Additionally, Hokkaido is reported to be affected by the long-range transport of organic matter from the Asian continent^27^. Here, the contributions of fractionated OA to total light absorption are assessed to understand the effects of organic aerosols over forest regions.

## Results and discussion

### Atmospheric concentrations and possible sources of OA factions.

**Figure 1:**
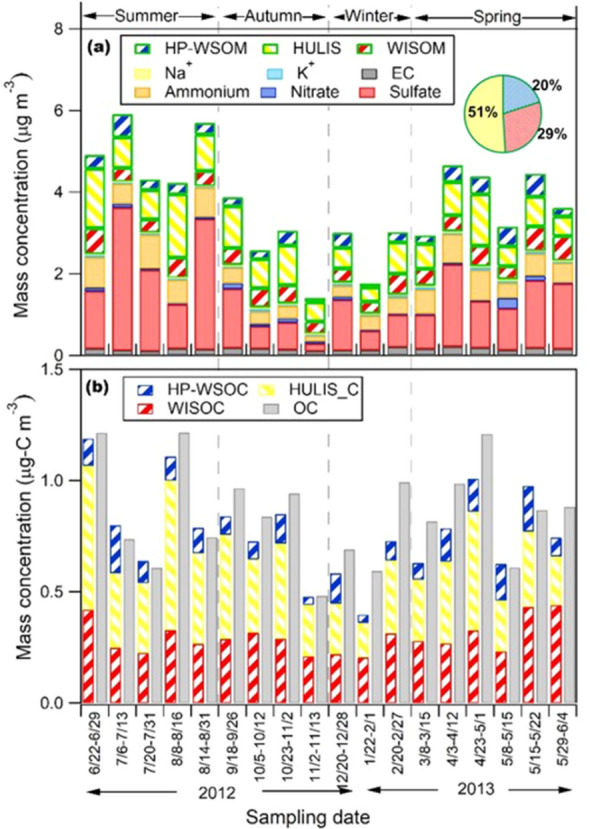
(**a**) Concentrations of extracted organic fractions (WISOM, HULIS, and HP-WSOM) and inorganic components in PM_0.95_ over TOEF. The pie chart represents the mean mass percentages of the relative contribution of the fractions. (**b**) Concentrations of organic carbon in the extracted fractions and total organic carbon in PM_0.95_. The format of the sampling date is start month/date to end month/date.

The atmospheric concentrations of extracted organic carbon (EOC) accounted for 90 ± 13% of the concentrations of total OC (Section S1 and Fig. 1b), showing that almost all the organics in PM_0.95_ were extracted in this study. The mass concentrations of the extracted organic fractions HULIS, HP-WSOM, and WISOM) and inorganic components in PM_0.95_ are presented in Fig. 1a. The annual and seasonal mass concentrations of all the are summarized in Supplementary Table S4. The mass concentrations of the extracted organic matter (EOM), which is the sum of the three fractions (HP-WSOM, HULIS, and WISOM), from all samples were 1.56 ± 0.45 μg m^−3^ (mean ± SD).

The studied aerosol can be characterized by relatively large proportion of HULIS. HULIS was the most abundant among the extracted organic fractions for all the samples. The mass concentration of HULIS was 0.81 ± 0.34 μg m^−3^, and those of WISOM and HP-WSOM were 0.44 ± 0.11 and 0.31 ± 0.14 μg m^−3^, respectively. HULIS were 51 ± 9% of the total EOM, followed by WISOM (29 ± 7%) and HP-WSOM (20 ± 8%). On the carbon basis, HULIS fraction was also the most abundant among the three fractions (Supplementary Table S4). The mean concentration of HULIS carbon (HULIS-C) corresponded to 30–61% of the concentration of EOC and 58–87% of the water-soluble organic carbon (WSOC) concentration. The abundance of HULIS-C relative to total OC from this study was comparable to the upper end in a background rural site in Hungary (38–72%), the central Tibetan Plateau (38–59%) and a pasture site in the Amazon rainforest (43–45%) but higher than a mountain forest park in China (8–13%), where HULIS in the referred studies were extracted by the SPE method using Oasis HLB columns^8,28–30^.

**Figure 2:**
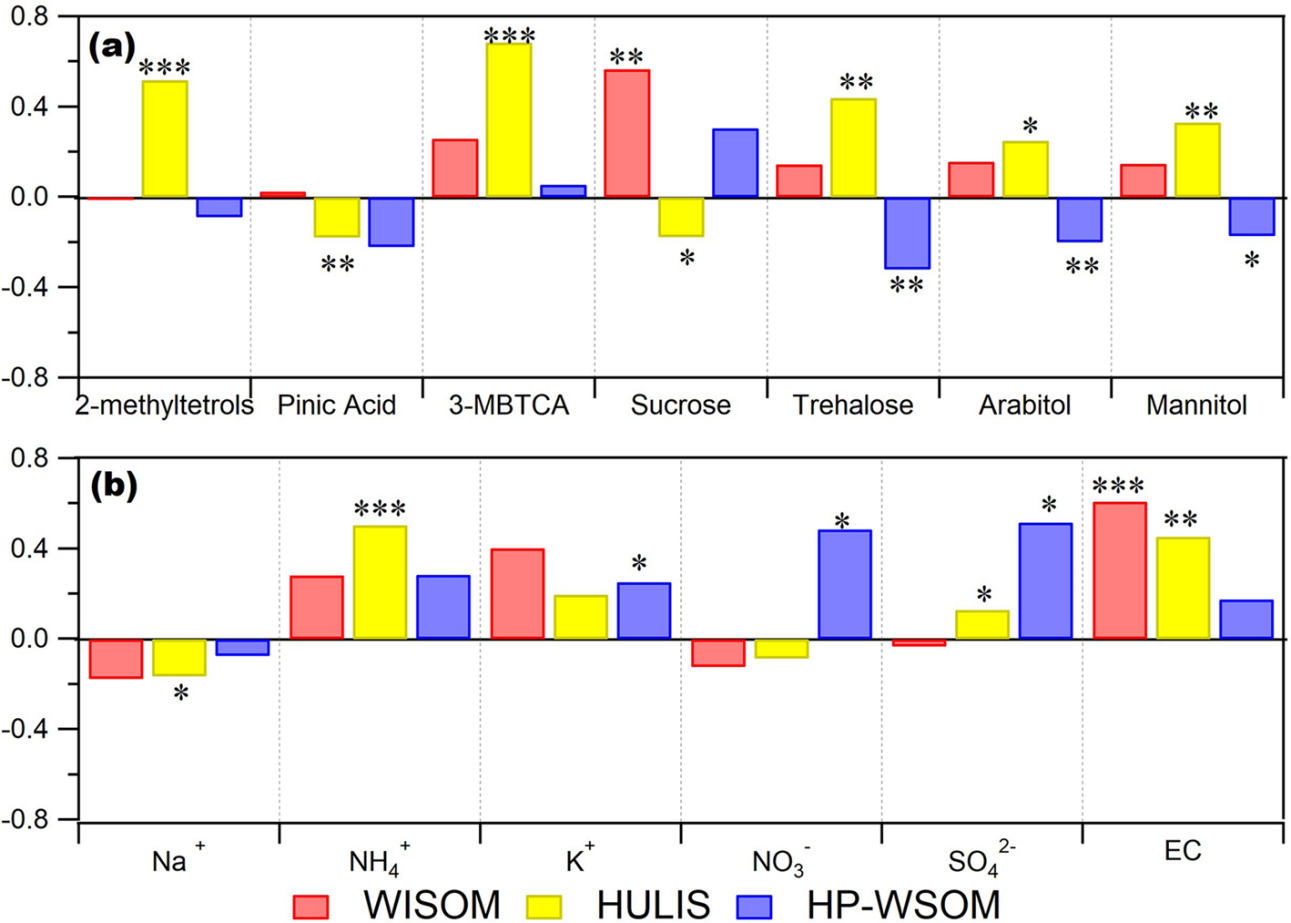
Pearson’s correlation coefficients (*n* = 18) and significance levels (*: < 0.05, **: < 0.01, ***: < 0.001) from the correlation analysis of the mass concentrations of WISOM, HULIS, and HP-WSOM and those of (**a**) biogenic molecular tracers and the analysis of (**b**) inorganic ions and EC.

BSOA could be a major source of HULIS in the studied area. The mass concentration of HULIS was on average highest in summer (Supplementary Table S4). The mean concentration of HULIS in summer was twice of that in winter (mean: 1.07 and 0.54 μg m^−3^, respectively). In addition, the mean proportion of HULIS among the three OA fractions in summer (58%) was also higher than that in winter (46%) (Supplementary Fig. S2). The highest contribution of HULIS to the mass concentration of OA in summer might be due to its enhanced photochemical formation. HULIS mass concentrations correlated with those of 3-methyl-1,2,3-butanetricarboxylic acid (3-MBTCA) (*n* = 18; *r* = 0.68) (Fig. 2 and Supplementary Fig. S3), which is a highly oxidized compound of *α*-pinene and is thus recognized as a tracer of *α*-pinene SOA. HULIS mass concentrations also correlated with those of 2-methyltetrols (the sum of 2-methylerythritol and 2-methylthreitol) (*n* = 18; *r* = 0.52) (Fig. 2 and Supplementary Fig. S4), which is a tracer of isoprene-SOA. These results indicates that BSOA have significant contribution in the mass concentration of HULIS. The contribution of BSOA to HULIS is also consistent with previous studies. The emissions of biogenic volatile organic compounds (BVOC) were reported to be highest in summer and autumn although it is for boreal forest^32^. In addition, biogenic emissions from vegetation in the background atmosphere in continental regions was reported to form atmospheric HULIS after oligomerization and photosensitization^7,31^.

In contrast with HULIS, WISOM showed a relatively small seasonal variation. The mass concentrations of WISOM were correlated with those of sucrose (*n* = 18; *r* = 0.57) (Fig. 2 and Supplementary Fig. S3), which has been suggested as a tracer of pollen grains^33^. The mass concentrations of WISOM also correlated well with those of EC (*n* = 18; *r* = 0.61) and moderately correlated with those of K^+^ (*n* = 18; *r* = 0.40), which are considered indicators of combustion and biomass burning, respectively (Supplementary Fig. S4). The correlation of WISOM with BSOA traces, such as 3-MBTCA and 2-methyltetrols, were weak and absent (*n* = 18; *r* = 0.26 and *r* = 0.01), respectively. TOEF is reported to be influenced by anthropogenic emissions from Tomakomai city and the industrial area to the south of the forest^25^. Hence, the major sources of WISOM would include natural primary emissions, biomass burning, and other anthropogenic activities while the contribution of BSOA should be minor.

The seasonal variation of HP-WSOM was slightly stronger than that of WISOM but not more than a factor of 1.5. The relationship of the mass concentration of HP-WSOM with biogenic molecular tracers and inorganic ions is presented in Fig. 2. The mass concentration of HP-WSOM correlates well with that of SO_4_^2−^ (*n* = 18; *r* = 0.54) (Supplementary Fig. S4). A previous study has shown that the highly oxygenated SOA of WSOM, where SOA was formed via aqueous-phase oxidation, correlates well with sulfate^34^. In this study, HP-WSOM might also be highly oxygenated SOA of WSOM, formed by aqueous-phase oxidation and transported to TOEF. The highly oxygenated nature of HP-WSOM described in the next section supports this explanation.

### Chemical structural characteristics

**Figure 3:**
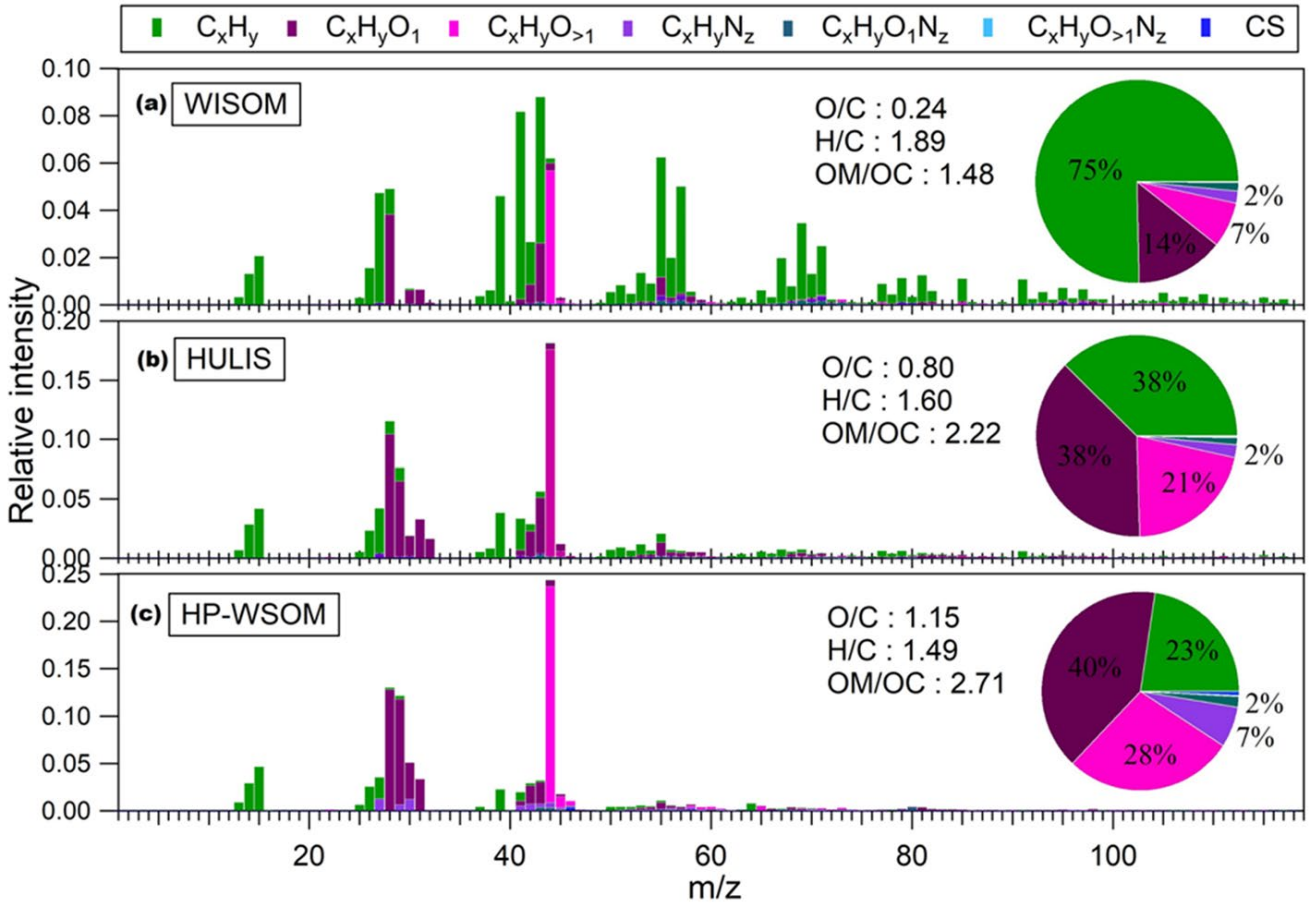
Normalized HR-AMS spectra of (**a**) WISOM, (**b**) HULIS, and (**c**) HP-WSOM. The pie charts represent the mean percentages of the ion groups in the spectra of the OA fractions.

The chemical characteristics of the three extracted OA fractions were obtained based on their HR-AMS spectra. Figure 3 presents the average HR-AMS spectra for the OA fractions. The proportions (%) of different fragment groups from the average HR-AMS spectra for each fraction in different seasons are listed in Table S5.

In the HR-AMS spectra of HULIS and HP-WSOM, CHO (C_x_H_y_O_1_ + C_x_H_y_O_>1_) groups showed high contribution, whereas WISOM consisted mainly of C_x_H_y_ fragments. Here, the CHO groups should represent oxygenated structures, such as, carboxylic acids, carbonyl compounds (ketones/aldehydes), alcohols, and esters and the C_x_H_y_ groups represent hydrocarbon-like structures. In HULIS, the fractional contribution of CHO groups was on average 59% The contribution of C_x_H_y_O_>1_ group was higher in summer (mean: 22%) than that in other seasons. This high contribution of oxygenated groups might be due to the photochemical formation of biogenic secondary OA (BSOA) that enhanced in summer. In HP-WSOM, the contributions of fragment ions containing oxygen were larger (mean: 68%) than that of HULIS. The C_x_H_y_O_1_ and C_x_H_y_O_>1_ groups on average contributed 40% and 28% of the spectra, respectively, with no remarkable seasonal change.

The C_x_H_y_ fragments accounted for 75% of WISOM, revealing that hydrocarbon-like structures were predominant in WISOM. The contribution of the C_x_H_y_O_>1_ group was enhanced in winter (mean: 10%). The increase of oxygenated groups in winter might be due to the enhanced contributions of the long-range transport of air masses. The five-day backward trajectories analysis using the Hybrid Single Particle Lagrangian Integrated Trajectory (HYSPLIT) model version 4 indicates that the influence of OA from the northeast Asian continent over TOEF in winter (Supplementary Fig. S5)^35–39^. The long-range transport of air masses in northern Japan was also reported in other studies where Asian winter monsoon transported OA containing air masses from Siberia and northeast China^27^.

The O/C ratios of WISOM, HULIS, and HP-WSOM were 0.24 ± 0.05, 0.80 ± 0.06, and 1.15 ± 0.10 (mean ± SD), respectively. The elemental ratios are presented in the van Krevelen diagram in Supplementary Fig. S6 and summarized in Supplementary Table S6. The O/C ratios of HULIS and HP-WSOM further indicate the highly oxygenated nature of these fractions and possible secondary formation, whereas the low O/C ratio of WISOM indicate its correspondence to primary OA^40^. The O/C ratios were highest in summer for HULIS (mean: 0.83) and highest in winter for WISOM (mean: 0.30), which is in accordance with the results from oxygen-containing ion groups in this study. As in the case of the O/C ratio, HP-WSOM had the highest OM/OC ratios (mean: 2.7), and WISOM had the lowest ratios (1.5) (Supplementary Table S6). The mean O/C ratios increased with the increase of polarity of the OA fractions as expected from the extraction procedure. The calculated densities of HULIS, HP-WSOM, WISOM, and EOM were 1441 ± 54, 1659 ± 72, 1013 ± 38, and 1317 ± 66 kg m^−3^ (mean ± SD), respectively.

### Light absorption properties

**Figure 4:**
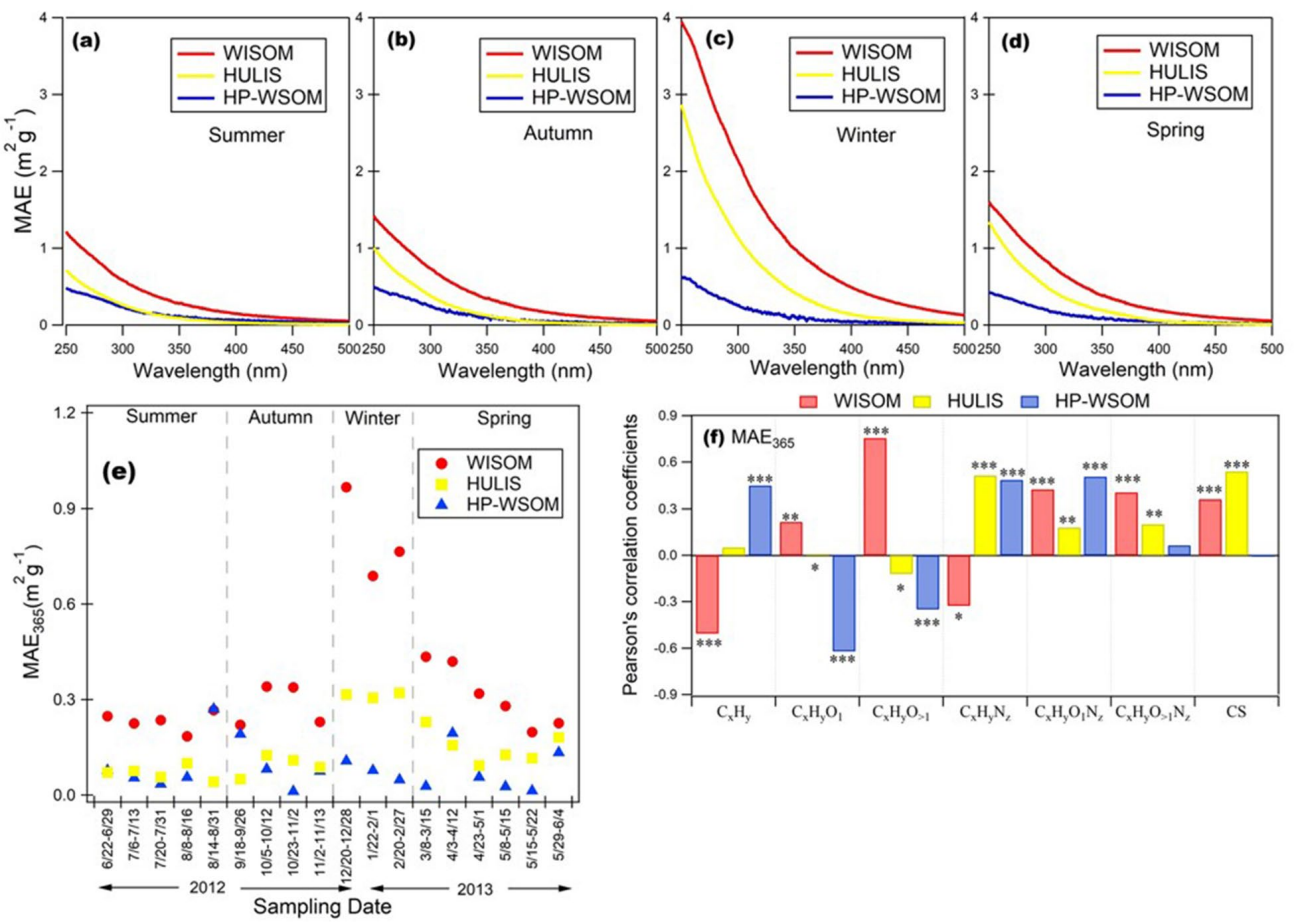
(**a–d**) Seasonal averages of the mass absorption efficiency (MAE) of the aerosol extracts, (**e**) temporal variations in MAE at 365 nm, and (**f**) Pearson’s correlation coefficients (*n* = 18) and significance levels (*: < 0.05, **: < 0.01, ***: < 0.001) from the correlation analysis of the MAE_365_ of WISOM, HULIS, and HP-WSOM and the relative intensities of HR-AMS-derived ion groups. The format of the sampling date is start month/date to end month/date.

The mass absorption efficiency (i.e., MAEs), also called the mass absorption coefficient (MAC), of all the OA fractions was calculated using Eq. (2). The seasonal averages of MAEs for WISOM, HULIS, and HP-WSOM are plotted in Fig. 4a–d and Supplementary Table S7. Generally, the absorption spectra and MAE of all OA extracts decrease monotonically with increasing wavelength. This characteristic is similar to that from other studies^4,23,41^. The MAEs at 365 nm (MAE_365_) of EOM throughout the study period was 0.21 ± 0.13 m^2^ g^−1^. MAE_365_ for WISOM and its carbon mass (calculated using the AMS-derived OM/OC ratios) were highest among all the extracts for all samples (mean ± SD: 0.37 ± 0.22 m^2^ g^−1^, corresponding to 0.55 ± 0.36 m^2^ g^−1^ C) (Fig. 4e and Supplementary Table S7), followed by HULIS (0.14 ± 0.09 m^2^ g^−1^, corresponding to 0.32 ± 0.21 m^2^ g^−1^ C) and HP-WSOM (0.09 ± 0.07 m^2^ g^−1^, corresponding to 0.23 ± 0.18 m^2^ g^−1^ C). The MAE_365_ of the OA extracts decreased with the increase of their polarity and O/C ratios, as reported for Nagoya urban aerosols^23^.

For WISOM, the MAE_365_ was, on average, also lower than that at highly polluted urban sites in Xi’an and Beijing (mean ± SD: 1.5 ± 0.5 m^2^ g^−1^ C and 1.5 ± 0.4 m^2^ g^−1^ C, respectively) and comparable with that in Nagoya (mean ± SD: 0.37 ± 0.13 m^2^ g^−1^) ^23,24^. In Beijing and Xi’an the high MAE_365_ were reported to be associated with anthropogenic emissions, e.g., primary vehicle emissions, and biomass burning activities especially from domestic heating in winter^18,42^. The differences in MAE_365_ in this study from those in the highly polluted urban areas might be due to the differences in the origins of the light absorbing WISOM.

**Figure 5:**
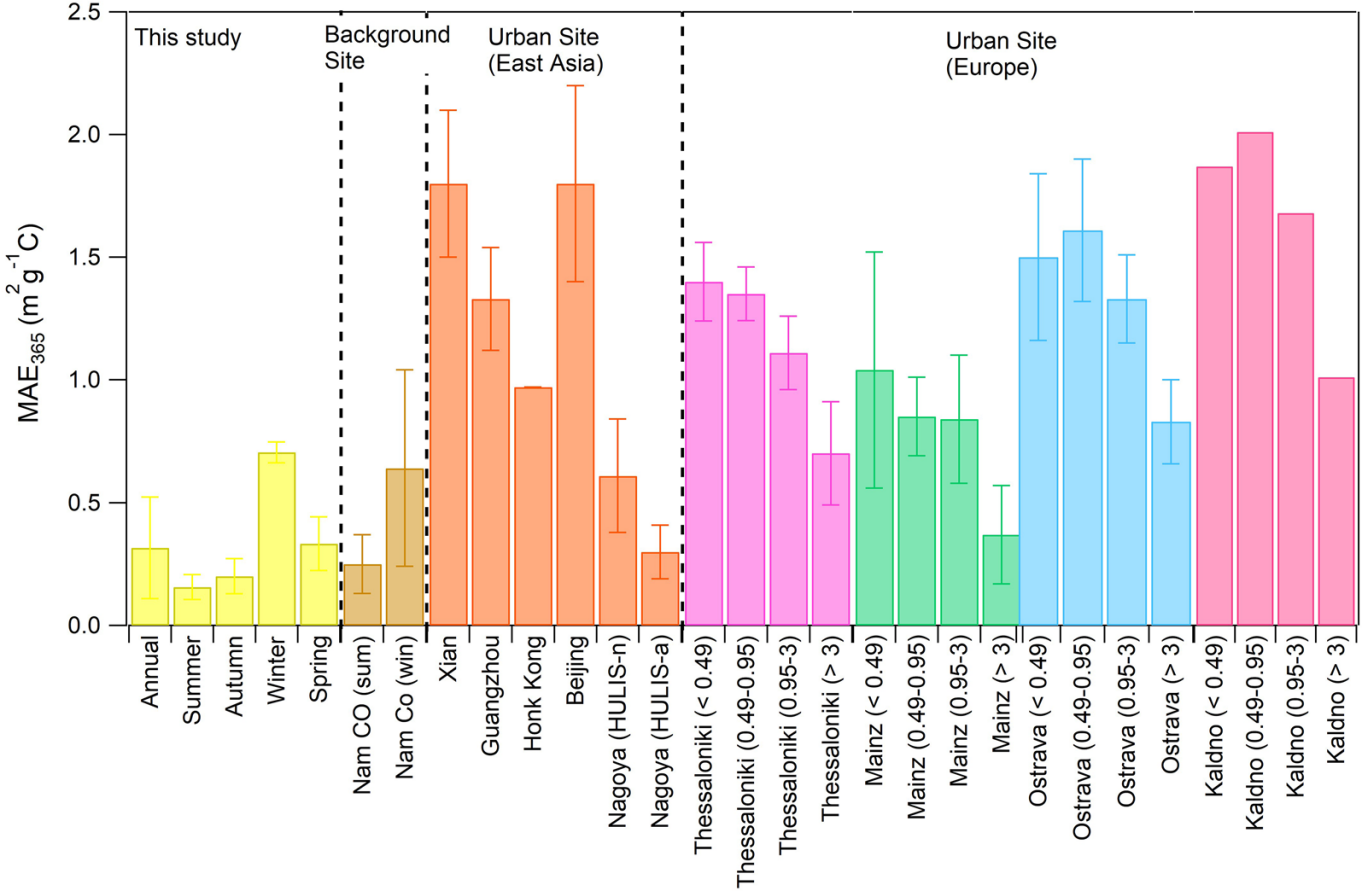
Comparison of MAE_365_ of HULIS from this study with the values at a background site^30^, urban sites of East Asia^23,24,37,38^ and urban sites of Europe^39^. The bar indicates the standard deviation.

The MAE_365_ of HULIS was also lower than those in urban sites. Comparison with previous studies (Fig. 5) shows that the MAE_365_ of HULIS was comparable with those in Nam Co station (Central Tibetan Plateau), where the annual aerosol optical depth was low^30^. The low MAE_365_ characteristics of the HULIS in this study are explained by the large contribution of BSOA because previous studies on BSOA showed that those generated under low NO_x_ conditions have insignificant light absorptivity in the wavelength region higher than 300 nm^2,46,47^. Similar to that in Nagoya, Xi’an, and Beijing, HP-WSOM on average showed a relatively lower MAE_365_ value than the values for HULIS and WISOM^23,24^. The MAE_365_ of HP-WSOM was also comparable with the average in Nagoya (mean ± SD: 0.11 ± 0.05 m^2^ g^−1^ C)^23^.

Comparison of the seasonal averages of MAEs of HULIS and WISOM (Fig. 4a–d) showed that the MAEs in winter, for wavelengths from 250 to 350 nm, were markedly higher than those in the rest of the seasons. The MAE_365_ of HULIS in winter was on average four times of that in summer. Previous researches suggests that anthropogenic activities in winter contributes to the high MAEs of HULIS and WISOM^23,24^. The long-range transport of aerosols from Asian continent may have contributed to the high MAEs of HULIS and WISOM in winter: biomass burning, including agricultural waste burning, and domestic use of coal combustion occurred during winter in northeast China, and Russian far-east might be the sources for OA with high MAEs^34,48,49^. The transport of air masses from northern Asia identified from the trajectory analysis in this study (Supplementary Fig. S5), and also a previous report about the influence of long-range transported organic matter over Hokkaido^27^ also supports this inference. The higher MAE values in winter than in summer has been reported at the background site in the Tibetan Plateau and a rural site in northern China, at both of which the enhanced values were correlated with biomass burning aerosols after long-range transport^10,30^. Anthropogenic emissions in Hokkaido, such as those from residential heating, may also have contributed to the high MAEs of HULIS and WISOM in winter.

The correlations between ion groups from HR-AMS and MAE_365_ are presented in Fig. 4f and Supplementary Fig. S7. The MAE_365_ values of HULIS were positively correlated with the C_x_H_y_N_z_ and CS ion group intensities. This result suggests that N- and S-containing organics contributed to the total light absorption of HULIS in TOEF. Chen et al.^23^ reported that O- and N-containing organics had larger contributions to light absorption in the case of HULIS over Nagoya. Organosulfates were found to be an important class of HULIS and formed through oligomerization^50,51^. Chamber studies also showed that organics containing S atom (organosulfate SOA) formed by the ozonolysis of anthropogenic and biogenic VOCs potentially absorb light^52,53^. Therefore, the relatively low abundance of organic compounds with N and S in HULIS in summer (Supplementary Table S5) may have contributed to the low MAE in this season. The MAE_365_ values of WISOM were positively correlated with C_x_H_y_O_>1_, C_x_H_y_ON_z_, C_x_H_y_O_>1_N_z_ and CS, suggesting that organic compounds with O, N and S atoms contributed to WISOM light absorption. This result is similar to the inference from our previous work that WISOM with O- and N-containing large aromatic molecules, and charge transfer complexes have large contribution to the light absorption over Nagoya City^54^. For HP-WSOM, positive correlations with C_x_H_y_N_z_ and C_x_H_y_ON_z_ suggest that its light absorption was largely contributed by the organic compounds with O and N atoms, which is also similar to the inference for HP-WSOM over Nagoya^23^.

The imaginary component of the refractive index (*k*) was obtained for OA fractions and EOM using Eq. (4) (Supplementary Table S8). Supplementary Fig. S8 shows the comparison of the imaginary refractive index of HULIS from this study and the indices from previous studies. The *k* values for HULIS at 390 and 532 nm were on average 3.34 × 10^−3^ and 5.42 × 10^−4^, respectively, which were lower than the values for HULIS from previous studies^55^. At 365 nm, the *k* value for HULIS (mean: 5.95 × 10^−3^) was higher than the values of HP-WSOM (3.44 × 10^−3^) but lower than WISOM (1.09 × 10^−2^).

The ratio of MAE at 250 nm to MAE at 365 nm, commonly expressed as E_2_/E_3_, is used to represent the total aromaticity or molecular weight of aquatic humic substances^56^. The E_2_/E_3_ values of WISOM, HULIS and HP-WSOM were 5.1 ± 0.3, 10.1 ± 2.0 and 8.5 ± 5.2, respectively (mean ± SD) (Supplementary Table S9). The less polar fractions on average have higher values of E_2_/E_3,_ suggesting higher aromaticity and higher molecular weight of less polar fractions. Nagoya aerosol also showed a similar tendency in our previous study^23^.

The wavelength dependence of light absorption, expressed as absorption Ångström exponent (AAE) was calculated for all the OA fractions using Eq. (3). The average AAE values in the range of 250–500 nm for WISOM, HULIS, and HP-WSOM were 4.86 ± 0.31, 7.46 ± 1.66, and 3.83 ± 2.64 (mean ± SD), respectively (Supplementary Fig. S9 and Table S10). For HULIS, the AAE values are similar to the values from previous studies. For the wavelength range of 300 to 700 nm, the AAE values reported for HULIS in different locations (Nagoya, Tibetan Plateau, rural site in north China, Xi’an, Guanzhong) varied from 4 to 10^10,23,30,44,57^. The AAE values of WISOM were similar to those found in Nagoya^23^. The values for WISOM were also similar to those of the methanol soluble organic matter in other locations of southeast Asia, which ranged from 3 to 10^4,23,58–60^. The AAE values of HP-WSOM from this study should have large uncertainty. From the wavelength of 300 nm to onward, the absorbances of the HP-WSOM were similar to the absorbances measured for blank samples. Chen et al.^23^ pointed out that the use of a single AAE value for OA for the range from 300 to 600 nm leads to a large deviation in fitted curves from the actual absorption spectra. In this study, the AAE values were also calculated separately for the UV and visible regions (Supplementary Table S8). For HULIS and WISOM, the AAE values tended to increase as the wavelength ranges shifted to a longer wavelength side. This trend is similar to what was found in Nagoya^23^.

### Contribution of the OA fraction to total light absorption

**Figure 6:**
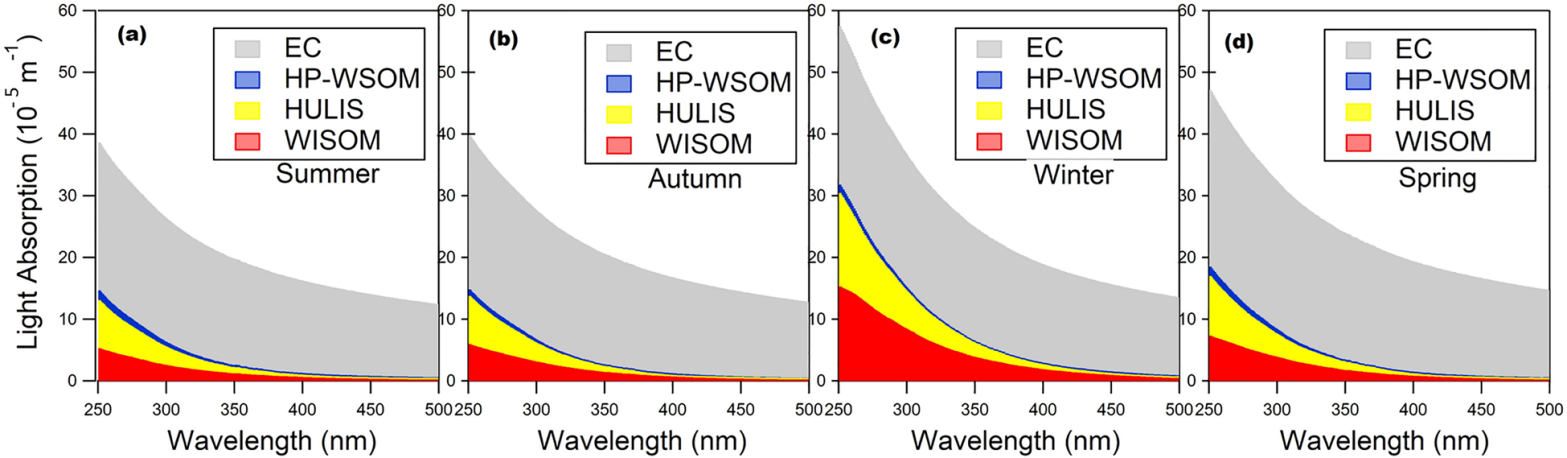
(**a–d**) Stacked plots of the seasonal averages of the contributions of the OA fractions, and EC to the total light absorption.

For the wavelength range from 250 to 500 nm, the contributions of EOM, OA fractions, and EC to the total absorption were estimated using Eq. (5). The contribution of EOM gradually decreased with increasing wavelength and accounted for 42–5% of the light absorption on average for all samples. The contribution of EOM was highest in winter. Figure 6a–d represents the seasonal averages of the contributions of the respective OA fractions and EC in the studied aerosol. In winter, the contribution of EOM in the range of 250–500 nm was 55–9% on average. The total light absorption by HULIS and WISOM in the range from 250 to 500 nm 87–92% of the light absorption by EOM on average for all samples. A large contribution of HULIS and WISOM to light absorption by EOM was also found in Nagoya^23^. At 365 nm, the light absorption of the EOM was on average 0.27 Mm^−1^, which was lower than that in Xi’an (65.4 Mm^−1^) and Beijing (42.1 Mm^−1^)^24^. This low light absorbing characteristics of forest aerosols compared to urban sites suggest that the contribution of OA to light absorption may have contrasting differences between forest and urban sites.

The average light absorption of EOM at 365 nm was, on average, 0.21 and 0.50 Mm^−1^ in summer and winter, respectively, which corresponded to 11% and 22% of the estimated total light absorption. Among the OA fractions, on average, the contribution of WISOM in the range of 250–500 nm tended to increase with increasing wavelength, while that of the HULIS fraction decreased (Supplementary Fig. S10). Saleh et al.^3^ showed that primary OA were more absorptive than secondary OA at long visible wavelengths, which supports the findings for WISOM in this study. The contribution of HP-WSOM was low and was 7–15% at 250–500 nm. The AAE values for the total light absorption of the aerosol (EC and EOM) for the range of 250–500 nm were 1.18 ± 0.10 (mean ± SD). The AAE values found at the study site based on sky radiometer measurements were 1.15 ± 0.84 in summer and 2.45 ± 0.91 in autumn for the wavelength range of 400–675 nm, which are similar to the values from the present study for summer (1.13 ± 0.02) but higher for autumn (1.16 ± 0.04)^26^. The difference may be because of the difference in the applied wavelength ranges and possible inhomogeneity of the vertical distribution of the aerosol.

The contribution of different OA fractions to the concentration and light absorption of nearly total OA (EOM) in forest aerosols was characterized in this study. BSOA contributed to HULIS, particularly in summer, and HULIS absorbed light less efficiently in summer. The relatively lower light absorbing characteristics of forest aerosols compared to urban sites are important because it supports the view that BSOA plays a role in negative climate feedback mechanisms^61,62^. In climate feedback mechanisms, BVOC emission from vegetation and subsequent BSOA formation increases with the increase of temperature which ultimately cool down the temperature by its negative radiative effect^61,62^. This role may become more important in the future because sulfate (SO_4_^2–^), which has a strong cooling effect on the climate, is expected to be less important in the near future as SO_2_ emissions decrease due to sulfur emission controls^63^. Our findings support the cooling effect of BSOA in forest regions, where the influence of anthropogenic pollution is low, in terms of the direct effect of aerosols on radiative forcing.

## Methods

### Aerosol sampling

Aerosol sampling was performed at Tomakomai Experimental Forest (TOEF) (42°42′N, 141°36′E) in Hokkaido Island, a site located in a cool-temperate zone. The aerosol samples were collected at ~ 18 m above the forest floor on quartz fiber filters (PALLFLEX Membrane Filter, 25 cm × 20 cm). Particles with diameters smaller than 0.95 µm were collected using a cascade impactor (Model TE-234, Tisch Environmental, Cleves, OH, USA) attached to a high-volume air sampler (Model 120SL, Kimoto Electric, Osaka, Japan). A total of 18 samples collected from June 2012 to June 2013 among a series of samples were used. Detailed information about the sampling is reported in Müller et al.^25^ and in Supplementary Table S1. The sample TMK-A-044 was in between late spring and early summer; if we consider it as a summer sample, the resulting average mass concentration of all the extracts change from 6 to 9% and the order of the seasonality does not change.

### Extraction and separation of HULIS and other aerosol components

A punched piece of PM_0.95_ samples with a diameter of 34 mm was used in this study to extract and fractionate OA components. At first, WSOM was extracted by ultrasonication using ~ 3.3 g of ultra-pure water (18 ΜΩ cm) three times, and then passed through a 0.2 µm pore syringe filter (PTFE, Millex-FG). After that, the WSOM was fractionated into two parts using one-step solid-phase extraction (SPE) described in Varga et al.^64^. An Oasis HLB column (6 cc, 200 mg; Waters) was used for SPE which was preactivated with 6 mL of methanol, followed by rinsing three times with 6 mL of water to remove residual methanol. The pH of the extract solution of WSOM was adjusted to 2 by adding 1 M HCl aqueous solution, and then the extract solution was passed through the Oasis HLB cartridge. After the solution of WSOM was passed through the column, it was rinsed with 1 mL of 0.01 M HCl solution. The organics in the effluent without adsorption were regarded as HP-WSOM. Then the cartridge was dried with N_2_ and 6 ml of methanol (MeOH) was used to elute the HULIS fraction. From the same filter punched, WISOM was extracted afterward by ultrasonication using ~ 3 g of MeOH and ~ 3 g of dichloromethane (DCM)/MeOH (2/1, v/v) mixture three times sequentially. The WISOM was then dried and redissolved in ~ 8 g of DCM/MeOH (2/1, v/v) mixture^11^. The extraction concentration of organic matter from samples in the solvent extraction procedure and the recovery of the solid-phase extraction are presented in section S1.

### HR-AMS analyses

A high-resolution time-of-flight aerosol mass spectrometer (HR-ToF-AMS, Aerodyne Research) was used to obtain the mass spectra of the OA tractions. The aerosol extracts were nebulized using compressed dry air and produced aerosol was passed through silica gel and activated carbon to remove solvent vapors. Then, the air of the aerosol was exchanged with Ar (purity: 99.99%) through a gas exchange device. The aerosol particles in the Ar flow were then analyzed using HR-ToF-AMS. The HR-AMS spectra acquired in the highly sensitive V mode were used to quantify the atmospheric concentrations of the OA fractions and to analyze ion groups. The W mode data were not used because the peaks were not well fitted. Three or four runs were used to obtain the average mass spectra for the extracts from respective aerosol samples. The atmospheric concentrations of the OA fractions were quantified by using phthalic acid as an internal standard^65,66^. The HR-AMS spectra were analyzed using Squirrel v.1.62A and Pika v1.22A software from http://cires.colorado.edu/jimenezgroup/ToFAMSResources/ToFSoftware/. All the ions were classified into C_x_H_y_, C_x_H_y_O_1_, C_x_H_y_O_>1_, C_x_H_y_O_1_N_z_, C_x_H_y_N_z_, C_x_H_y_O_>1_N_z_, and CS groups. The quality control of HR-AMS analysis is presented in section S2. Elemental analysis was performed to determine the O/C, H/C, and OM/OC ratios. The concentrations based on carbon were calculated using the OM/OC ratios. The density of OA fractions was estimated by the method in Kuwata et al.^67^.

### Analysis of carbonaceous components, inorganic ions and biogenic molecular tracers

The atmospheric concentrations of organic carbon (OC) and elemental carbon (EC) in the PM_0.95_ samples were obtained using an OC/EC carbon aerosol analyzer (Sunset Laboratory Inc.) with the IMPROVE_A temperature protocol.

An organic carbon analyzer (Model TOC-LCHP, Shimadzu) was used to determine the water-soluble organic carbon (WSOC) concentration for all the samples. The concentrations of inorganic ions (Na^+^, K^+^, NH_4_^+^, NO_3_^−^, and SO_4_^2−^) were analyzed using an ion chromatograph (Model 761 compact IC; Metrohm).

A capillary gas chromatograph coupled to a mass spectrometer (GC7890 and MSD5975C, Agilent) was used to analyze biogenic molecular tracers (trehalose, arabitol, mannitol, sucrose, 2-methyltetrols (the sum of 2-methylerythritol and 2-methylthreitol), 3-methyl-1,2,3-butanetricarboxylic acid (3-MBTCA), and pinic acid). The details of these methods are reported elsewhere^25^. For biogenic molecular tracers, the scaling factors for mass spectral signals to calculate their concentrations were not validated for this study because their concentrations were only used for correlation analysis.

### UV−visible absorption spectra

The UV–visible absorption spectra of the extracts were measured using a UV–visible spectrophotometer (V-570, JASCO). A 1-cm path length quartz cell was used to measure the spectra of all extracts and solvents. The spectra were measured for three times from 190 to 800 nm with an interval of 0.5 nm. The UV–visible absorption spectra of the solvents were subtracted from the OA fractions spectra. The light absorption coefficient (m^–1^) was calculated as^23^:

1 $$\mathrm{Abs}_{\lambda} = (\mathrm{A}_{\lambda} -\mathrm{ A}_{700})/l$$

where *λ* refers to the wavelength; *A*_*λ*_ and *A*_700_ are the absorbance at wavelengths *λ* and 700 nm, respectively; and *l* is the path length. The light absorption at 700 nm was very weak (absorbance: − 0.00071 to 0.00539) in this study.

The mass absorption efficiency (MAE, m^2^ g^–1^) of the organics in the OA fractions was calculated by^23^:

2 $$MAE_{\lambda} = Abs_{\lambda }/C_{\mathrm{OM}} \times \,ln\, (10)$$

where *Abs*_*λ*_ is the light absorption coefficient (m^–1^) at a wavelength of *λ* nm and *C*_OM_ is the organic mass concentration in the solution of OA fractions.

The MAEs of all the OA fractions were calculated for the wavelength range from 250 to 500 nm. The lower bound was set to 250 nm because ammonium sulfate and ammonium nitrate in aqueous solutions can absorb ultraviolet light in wavelengths shorter than 250 nm, and the specific absorbance cutoff wavelengths of the solvents DCM and MeOH were 235 nm and 210 nm, respectively^68,69^.

Another important optical parameter for atmospheric aerosol components, the absorption Angström exponent (AAE), was calculated by the following equation^23^:

3 $$MAE=a{\lambda }^{{-A}^{0}}$$

where *a* is a constant and *A*^0^ is the Ångström exponent.

The imaginary component of the refractive index (*k*) was estimated using the following equation^70^:

4 $$k =\frac{{\rho }_{OA}\times \lambda \times MAE}{4\pi } \times 10^{-3}$$

where *ρ*_OA_ is the OA density (in g cm^−3^).

The relative contributions of the OA fractions and EC to the total light absorption of each aerosol extract were estimated using the following equation without considering any kind of aerosol mixing states ^23^:

5 $$Abs{(\lambda )}_{total}=\sum MAE{(\lambda )}_{i}.{C}_{i}^{{\prime}}+MAE{(\lambda )}_{EC}.{C}_{EC}$$

where *C*^*′*^_*i*_ is the atmospheric concentration of the OA fraction *i*.

In this study, the MAE of EC was calculated according to Eq. (3) using the MAE of EC at 550 nm and its Å of 7.5 m^2^ g^−1^ and 1, respectively^71^.

## Supplementary Information

Supplementary Information 1.

Supplementary Information 2.

Supplementary Information 3.

Supplementary Information 4.

Supplementary Information 5.

## Data Availability

All the data to support the findings of this study are provided in the manuscript or the supporting information materials. Other data are available on request to the corresponding author.
