# Author Correction: Abundance, chemical structure, and light absorption properties of humic-like substances (HULIS) and other organic fractions of forest aerosols in Hokkaido

**DOI:** 10.1038/s41598-023-40936-6

**Published:** 2023-09-04

**Authors:** Sonia Afsana, Ruichen Zhou, Yuzo Miyazaki, Eri Tachibana, Dhananjay Kumar Deshmukh, Kimitaka Kawamura, Michihiro Mochida

**Affiliations:** 1 Graduate School of Environmental Studies, Nagoya University, Nagoya, 464‑8601 Japan; 2 Institute of Low Temperature Science, Hokkaido University, Sapporo, 060‑0819 Japan; 3 Chubu Institute for Advanced Studies, Chubu University, Kasugai, 487‑8501 Japan; 4 Institute for Space‑Earth Environmental Research, Nagoya University, Nagoya, 464‑8601 Japan; 5 Present Address: Institute for Space‑Earth Environmental Research, Nagoya University, Nagoya, 464‑8601 Japan; 6 Present Address: Space Physics Laboratory, Vikram Sarabhai Space Centre, Thiruvananthapuram, 695022 India

Correction to: *Scientific Reports* 10.1038/s41598-022-18201-z, published online 23 August 2022

The original version of this Article contained errors in the Introduction, Results and Discussion section, Figure 5 and 6, Acknowledgements section and in Supplementary Information 2 and 3.

In the Introduction,

“Chen et al.^24^ showed that in Nagoya city, WISOM had higher contribution to the total light absorption of OA than that of WSOM and was dominant in the visible region.”

now reads:

“Chen et al.^23^ showed that in Nagoya city, WISOM had higher contribution to the total light absorption of OA than that of WSOM and was dominant in the visible region.”

In the Results and Discussion section, under the subheading ‘Light absorption properties’,

“For WISOM, the MAE_365_ was, on average, also lower than that at highly polluted urban sites in Xi’an and Beijing (mean ± SD: 1.5 ± 0.5 m^2^ g^−1^ C and 1.5 ± 0.4 m^2^ g^−1^ C, respectively) and comparable with that in Nagoya (mean ± SD: 0.37 ± 0.13 m^2^ g^−1^)^23,24,30^.”

now reads:

“For WISOM, the MAE_365_ was, on average, also lower than that at highly polluted urban sites in Xi’an and Beijing (mean ± SD: 1.5 ± 0.5 m^2^ g^−1^ C and 1.5 ± 0.4 m^2^ g^−1^ C, respectively) and comparable with that in Nagoya (mean ± SD: 0.37 ± 0.13 m^2^ g^−1^)^23,24^.”

In the Results and Discussion section, under the subheading ‘Contribution of the OA fraction to total light absorption’,

“At 365 nm, the light absorption of the EOM was on average 2.7 Mm^−1^, which was lower than that in Xi’an (65.4 Mm^−1^) and Beijing (42.1 Mm^−1^)^24^.”

now reads:

“At 365 nm, the light absorption of the EOM was on average 0.27 Mm^−1^, which was lower than that in Xi’an (65.4 Mm^−1^) and Beijing (42.1 Mm^−1^)^24^.”

“The average light absorption of EOM at 365 nm was, on average, 2.1 and 5.0 Mm^−1^ in summer and winter, respectively, which corresponded to 11% and 22% of the estimated total light absorption.”

now reads:

“The average light absorption of EOM at 365 nm was, on average, 0.21 and 0.50 Mm^−1^ in summer and winter, respectively, which corresponded to 11% and 22% of the estimated total light absorption.”

In Figure 6, in the vertical axes “Light Absorption (10^−5^ m^−1^)” was incorrectly given as “Light Absorption (10^−6^ m^−1^)”. The original Figure 6 and accompanying legend appear below.

**Figure 6:**
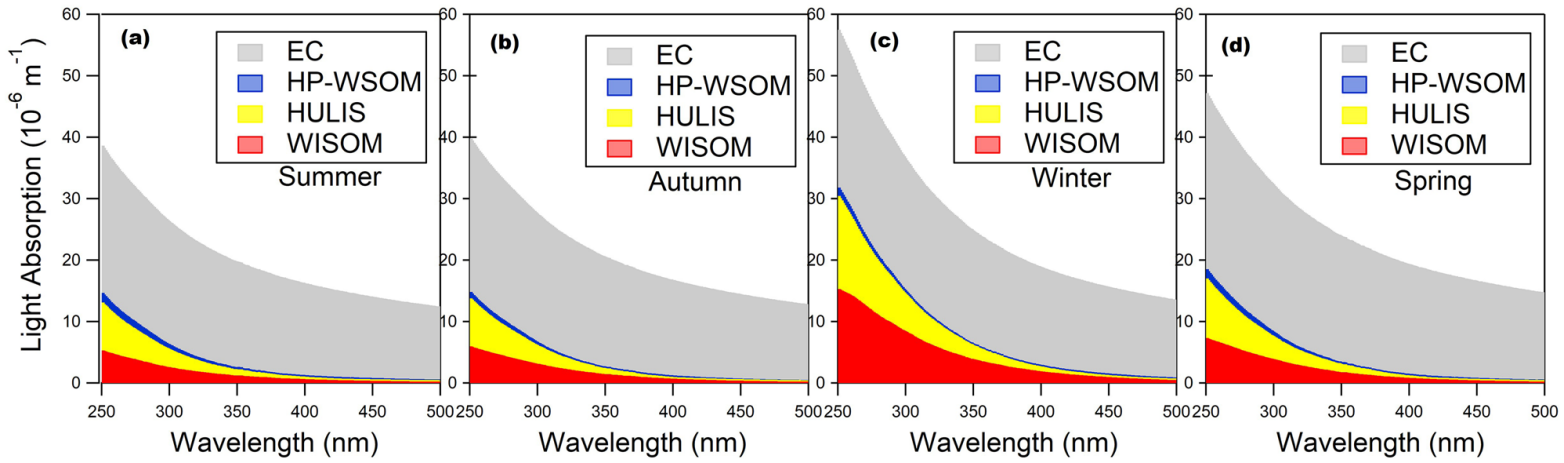
(**a–d**) Stacked plots of the seasonal averages of the contributions of the OA fractions, and EC to the total light absorption.

In the legend of Figure 5,

“Comparison of MAE_365_ of HULIS from this study with the values at a background site^30^, urban sites of East Asia^18,23,43,44^ and urban sites of Europe^45^. The bar indicates the standard deviation.”

To:

“Comparison of MAE_365_ of HULIS from this study with the values at a background site^30^, urban sites of East Asia^23,24,37,38^ and urban sites of Europe^39^. The bar indicates the standard deviation.”

The original version of this Article also contained an error in the Acknowledgements section.

“We would like to thank the Research Center for Materials Science, Nagoya University for the use of UV–vis spectrophotometer and Kin-ichi Oyama for the technical support. We would like to thank Prof. Tsutom Hiura for his contribution to the placement of the sampler and the management of TOEF. This study was supported by JSPS KAKENHI Grant Number JP19H04253 and JSPS under the Joint Research Program implemented in association with DFG (JRPs-LEAD with DFG, Grant Number: JPJSJRP20181601).”

now reads:

“We would like to thank the Research Center for Materials Science, Nagoya University for the use of UV–vis spectrophotometer and Kin-ichi Oyama for the technical support. We would like to thank Prof. Tsutom Hiura for his contribution to the placement of the sampler and the management of TOEF. This study was supported by JSPS KAKENHI Grant Number JP19H04253 and JP19KK0265 and JSPS under the Joint Research Program implemented in association with DFG (JRPs-LEAD with DFG, Grant Number: JPJSJRP20181601).”

In addition, the Supplementary Information file 2 and 3 published with this Article contained errors.

The original Supplementary Information files are provided below.

The original Article and accompanying Supplementary Information files have been corrected.

### Supplementary Information

Supplementary Information 2.

Supplementary Information 3.

