# Supplementary material for: Abundance, chemical structure, and light absorption properties of humic-like substances (HULIS) and other organic fractions of forest aerosols in Hokkaido
Source: Sci Rep. 2022 Aug 23;12:14379. doi: 10.1038/s41598-022-18201-z (PMC9399238; doi:10.1038/s41598-022-18201-z)
Supplement: Supplementary file 4 — Supplementary Information 4. [file 41598_2022_18201_MOESM4_ESM.pdf]

Supplementary Information

Abundance, chemical structure, and light absorption properties of humic-like substances (HULIS) and other organic fractions of forest aerosols in Hokkaido

Sonia Afsana[1], Ruichen Zhou[1,a], Yuzo Miyazaki[2], Eri Tachibana[2], Dhananjay Kumar Deshmukh[,3,b], Kimitaka Kawamura[3] and Michihiro Mochida[1,4,*]

[1]Graduate School of Environmental Studies, Nagoya University, Nagoya 464-8601, Japan

[2]Institute of Low Temperature Science, Hokkaido University, Sapporo, 060-0819, Japan

[3]Chubu Institute for Advanced Studies, Chubu University, Kasugai 487-8501, Japan

[4]Institute for Space-Earth Environmental Research, Nagoya University, Nagoya 464-8601, Japan

* Corresponding Author, Graduate School of Environmental Studies, Nagoya University, Nagoya 464-8601, Japan; Institute for Space-Earth Environmental Research, Nagoya University, Nagoya 464-8601, Japan; orcid.org/0000-0001-9557-5138;

Email: mochida@isee.nagoya-u.ac.jp

a. Present address: Institute for Space-Earth Environmental Research, Nagoya University, Nagoya 464-8601, Japan.

b. Present address: Space Physics Laboratory, Vikram Sarabhai Space Centre, Thiruvananthapuram 695022, India.

S1. Extraction efficiency and recovery of OM

To evaluate the extraction efficiency of organic matter from samples via the solvent extraction procedure, we compared the atmospheric concentrations of carbon in extracted OA fractions (HULIS, HP-WSOM, and WISOM) derived from analysis using HR-AMS and OC from thermal analysis (Figure S1a). The atmospheric concentrations of extracted organic carbon (EOC), i.e., the summation of organic carbon in respective fractions (EOC = HP-WSOC + HULIS carbon (HULIS_C) + WISOC) corresponded to 90 ± 13% (mean ± SD; n = 18; R^2 = 0.77) of atmospheric OA concentrations from the thermal analysis. The percentages are considered to represent the extraction efficiency on the assumption that the thermal analysis quantifies the exact amounts of OC. Here, the organic carbon concentration of each fraction was calculated from the OM/OC ratios obtained from the HR-AMS analysis.

The recovery of OA when utilizing HLB column was assessed as follows. The WSOM in the samples was divided into two fractions, one for SPE and the other for the quantification of WSOM. The fractions from SPE (HULIS and HP-WSOM) and WSOM were quantified using AMS. The recovery of compounds via SPE analysis using HLB column was then calculated as the sum of HULIS and HP-WSOM divided by WSOM (Table S2). The recovery ranged from 95% to 110%, indicating that almost all of the adsorbed compounds were eluted from HLB column by this method. The concentrations of carbon in the extracted OA fractions (HULIS and HP-WSOM) derived from the analysis using HR-AMS and WSOC from the TOC analyzer also correlated well (n = 18; R^2 = 0.63) (Figure S1b).

S2. Quality control of HR-AMS

The HR-AMS spectra were analyzed using Squirrel v.1.62A and Pika v1.22A software (http://cires.colorado.edu/jimenezgroup/ToFAMSResources/ToFSoftware/). In the analysis using Squirrel, one run number was detected as bad run and was eliminated from the to-do

wave to obtain the preprocessed data for further analysis. We considered possible interference of negative peaks not assigned to organics from Squirrel, for the initial V-mode run for the WISOM from sample TMK-A-023 (summed intensity of negative peaks (in Hz): 44% of that of organics); the run was included in the analysis because the exclusion of the run from the averaging of the spectra for the WISOM resulted in just a slight change (0.10% decrease) in the calculated atmospheric mass concentration value. For AMS analysis, three blank filters collected before, during and after a series of atmospheric samplings (Table S1) were extracted and analyzed in the same manner as the $PM_{0.95}$ samples. The blank levels were assessed with HR-AMS analysis and corresponded to 13%, 12%, and 18% of the lowest concentrations of the solutions of WISOM, HULIS, and HP-WSOM, respectively.

To assess the repeatability of the offline AMS analysis, three filter samples were extracted and analyzed in duplicate, and the relative standard deviation (RSD) was approximated as the mean of the standard deviation values. The RSDs of the AMS-derived concentrations of WISOM, HULIS, and HP-WSOM were 12.7%, 5.5%, and 11.7%, respectively. The estimated RSDs of the relative intensities of eight fragment groups (C_xH_y, $C_xH_yO_1$, $C_xH_yO_{>1}$, and $C_xH_yN_z$, $C_xH_yON_z$, $C_xH_yO_{>1}N_z$, CS) were in the range of 0.6–28.1% for WISOM, HULIS, and HP-WSOM. The CS group of WISOM and HP-WSOM showed high RSDs (30.7–52.5%), which might have been caused by the measurement uncertainty of CS, the abundance of which was low (< 1%) in WISOM and HP-WSOM.

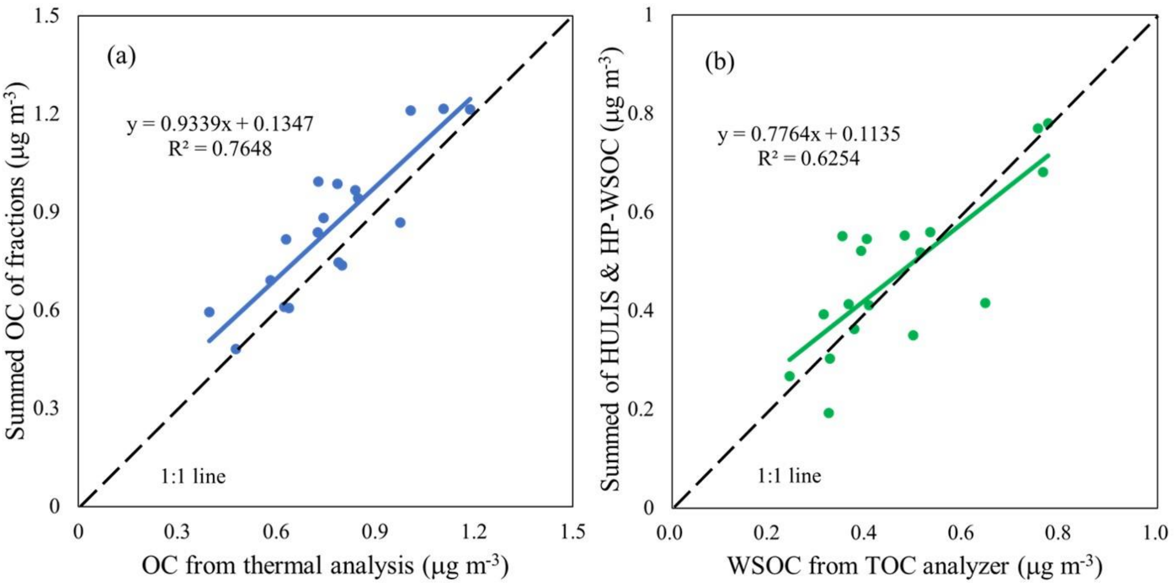

Figure S1. (a) Summed mass concentrations of carbon in extracted OA fractions (WISOM, HULIS, and HP-WSOM) from the HR-AMS analysis versus mass concentrations of OC from thermal analysis of filter samples. (b) Summed mass concentrations of carbon in HULIS and HP-WSOM from the HR-AMS analysis versus WSOC from TOC analyzer. The solid lines (blue and green) indicate the regression line, and the black dashed lines indicate the 1:1 line.

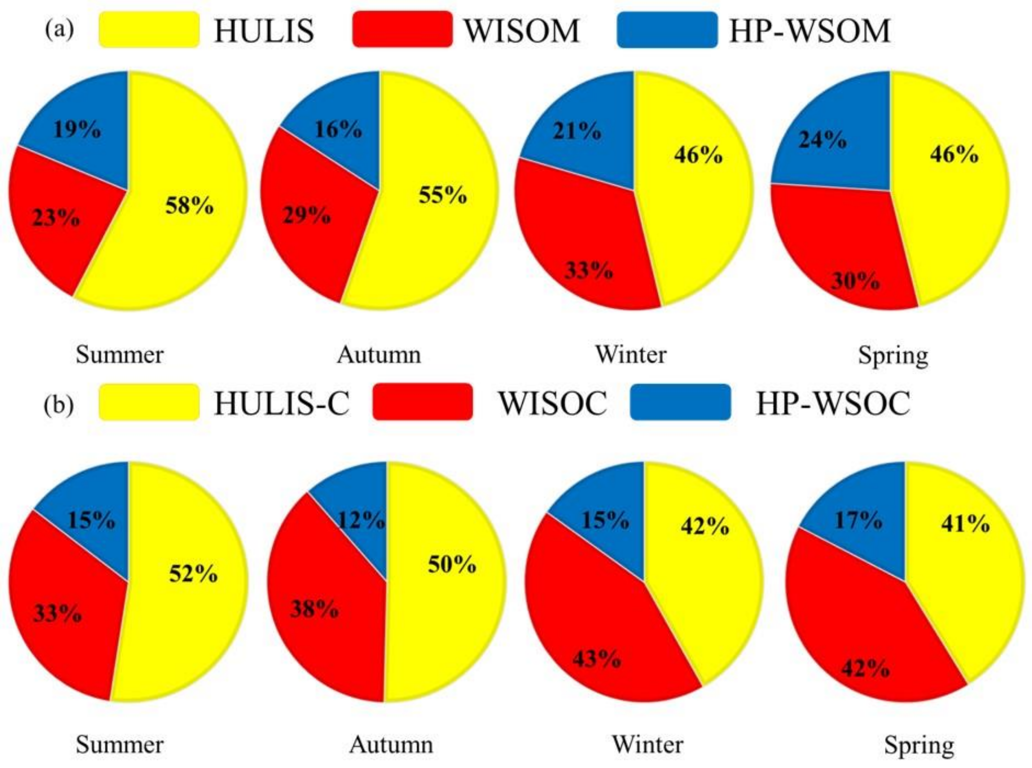

Figure S2. Seasonal mean mass percentages of the (a) OA fractions and (b) organic carbon in the OA fractions.

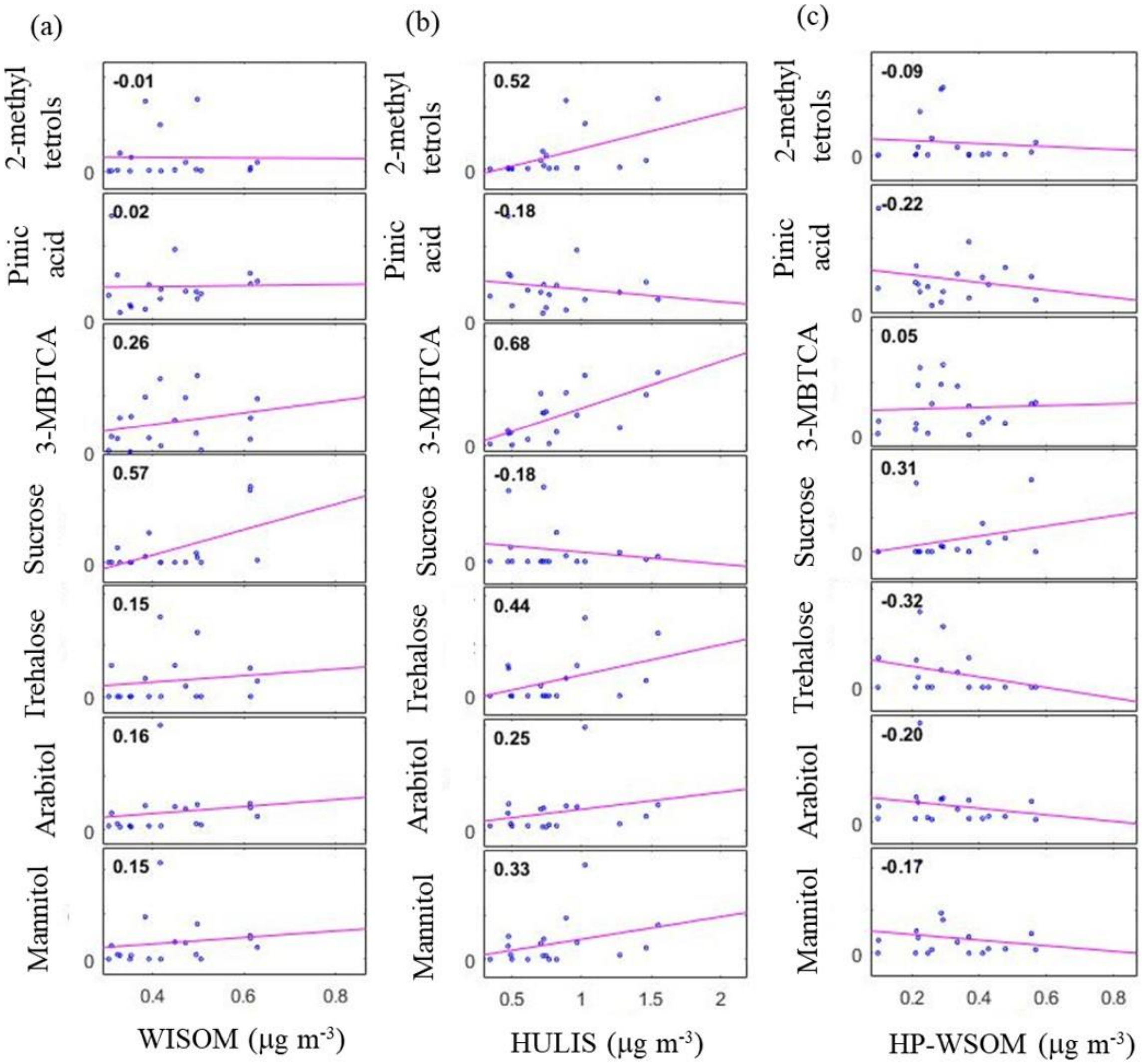

Figure S3. Concentrations of biogenic molecular tracers versus those of (a) WISOM, (b) HULIS, and (c) HP-WSOM. The purple lines indicate the regression lines, and the inset numbers are the Pearson's correlation coefficients. The concentrations of biogenic molecular tracers are in arbitrary units.

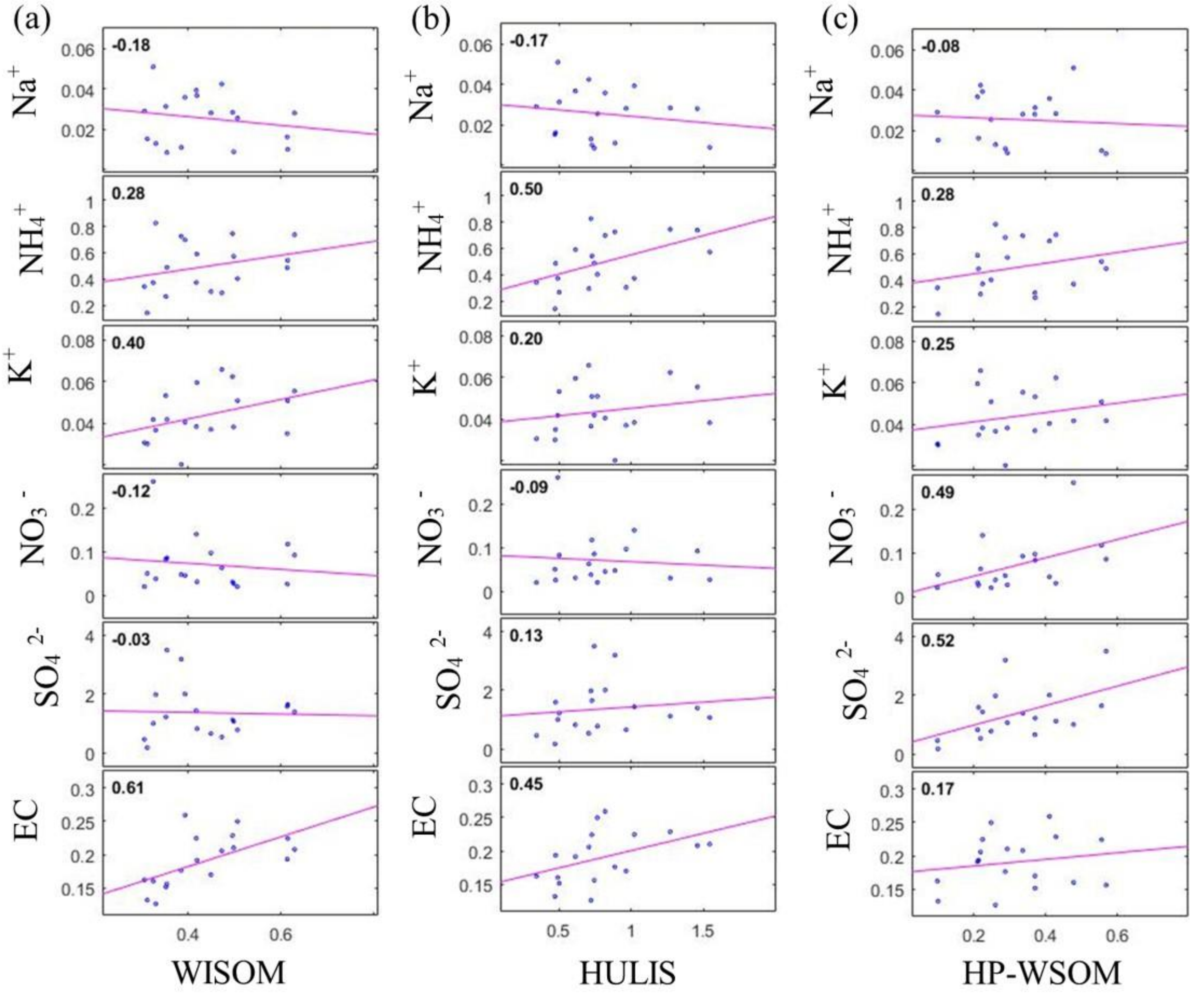

Figure S4. Mass concentrations of inorganic ions and EC versus those of (a) WISOM, (b) HULIS and (c) HP-WSOM (μg m^{-3}). The purple lines indicate the regression lines, and the inset numbers are the Pearson's correlation coefficients.

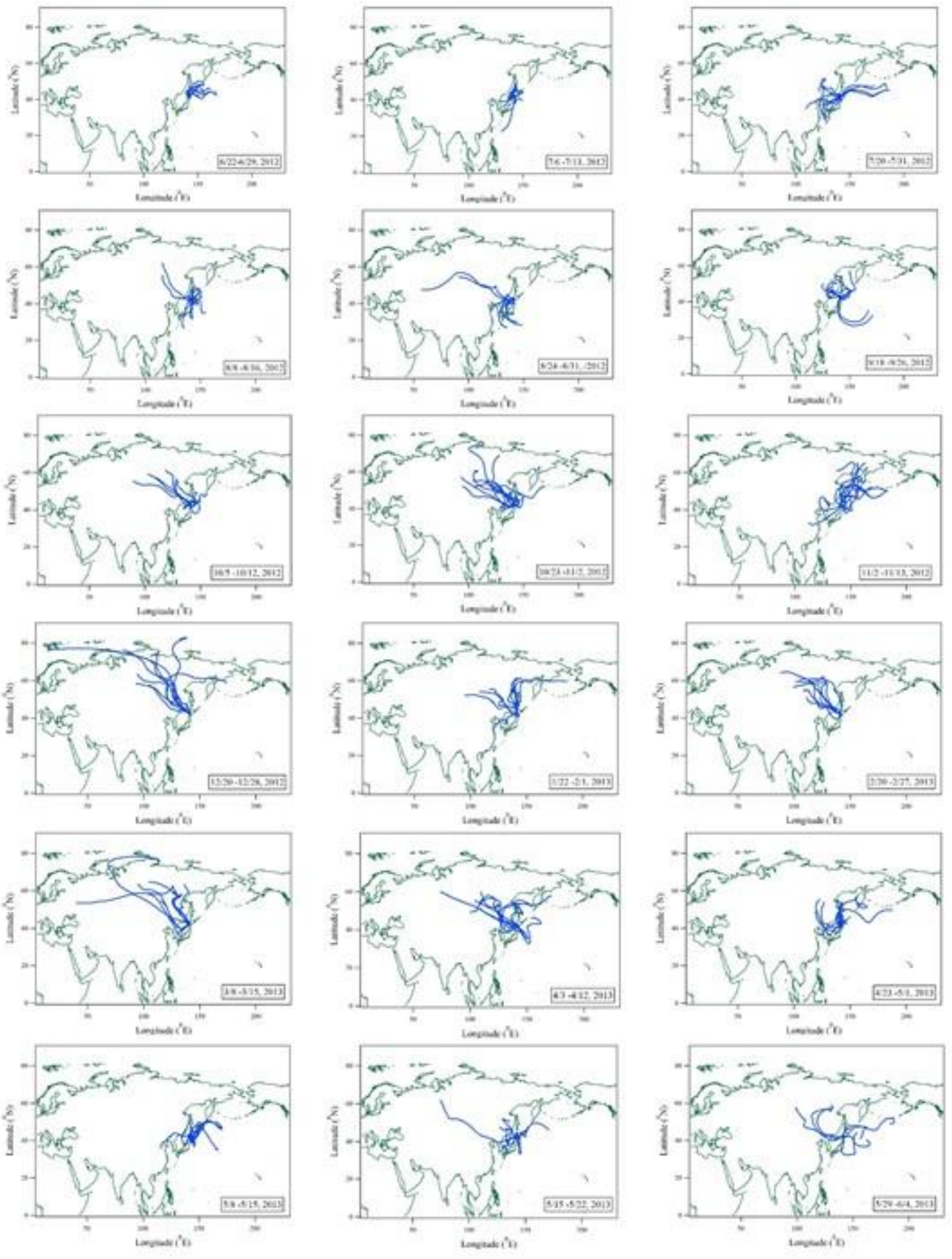

Figure S5. Backward trajectories of air masses for each sampling period (start time: 14:00 Japan Standard Time (every day); start height: 500 m above ground level; duration: 120 h).

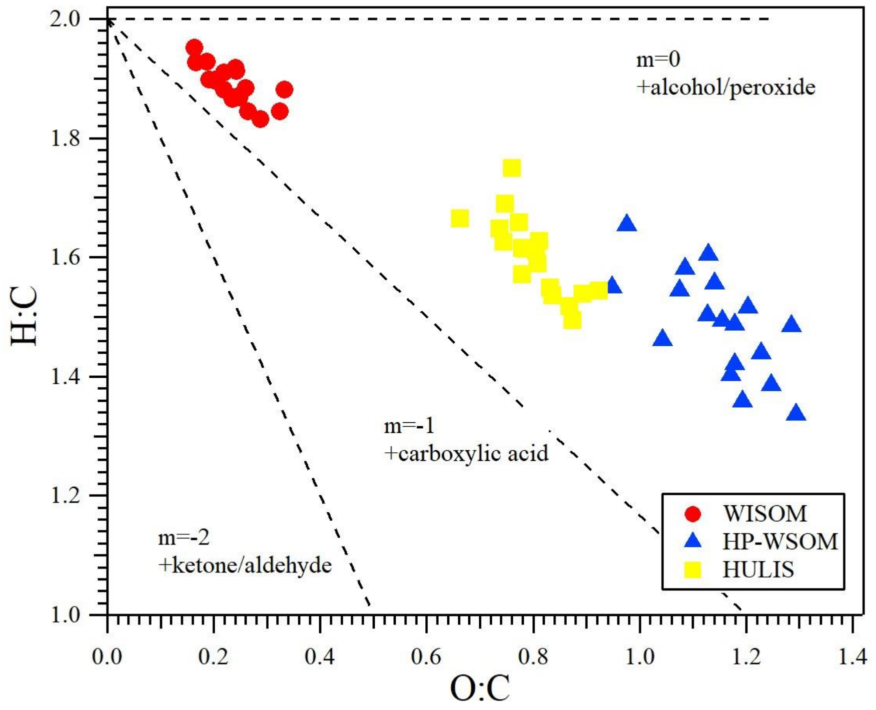

Figure S6. Van Krevelen diagram of HULIS, HP-WSOM, and WISOM from HR-AMS analysis.

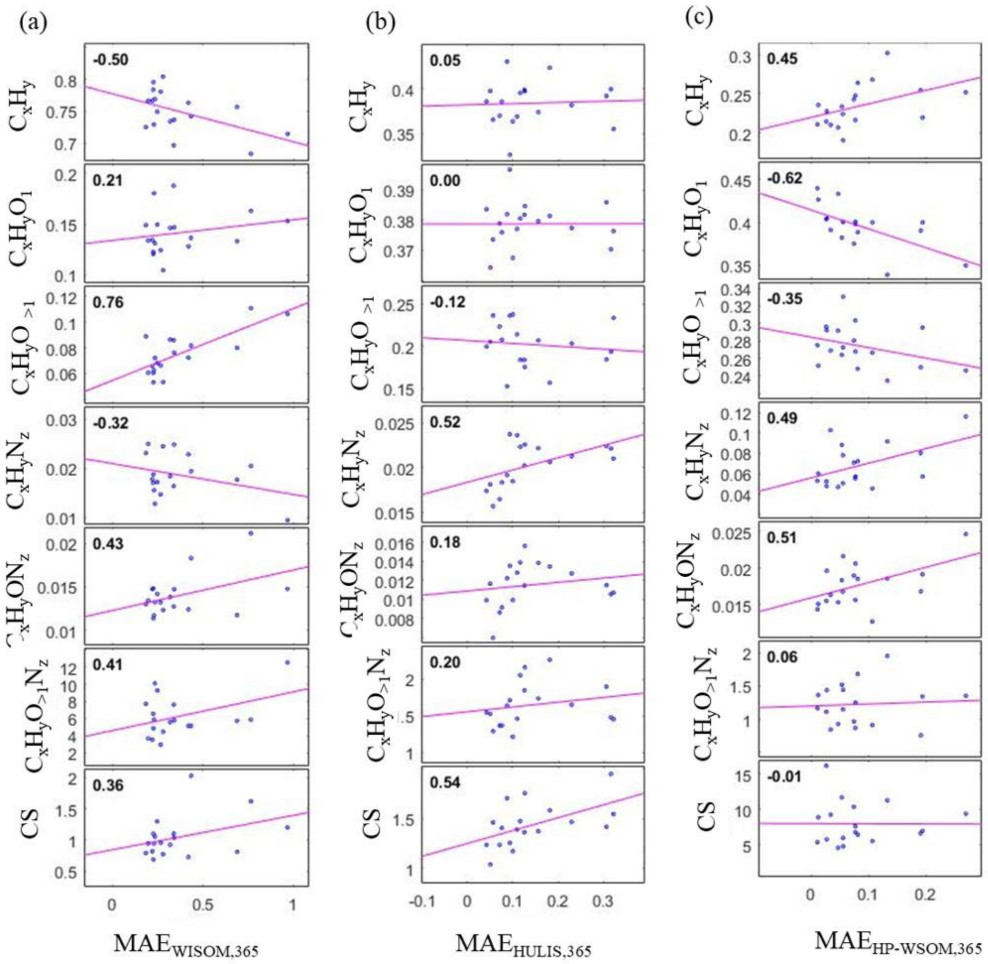

Figure S7. Mass absorption efficiency (MAE) at 365 nm (m^2 g^{-1}) versus relative intensities of ion groups from the HR-AMS spectra for (a) WISOM, (b) HULIS, and (c) HP-WSOM. The purple lines indicate the regression lines, and the inset numbers are the Pearson's correlation coefficients.

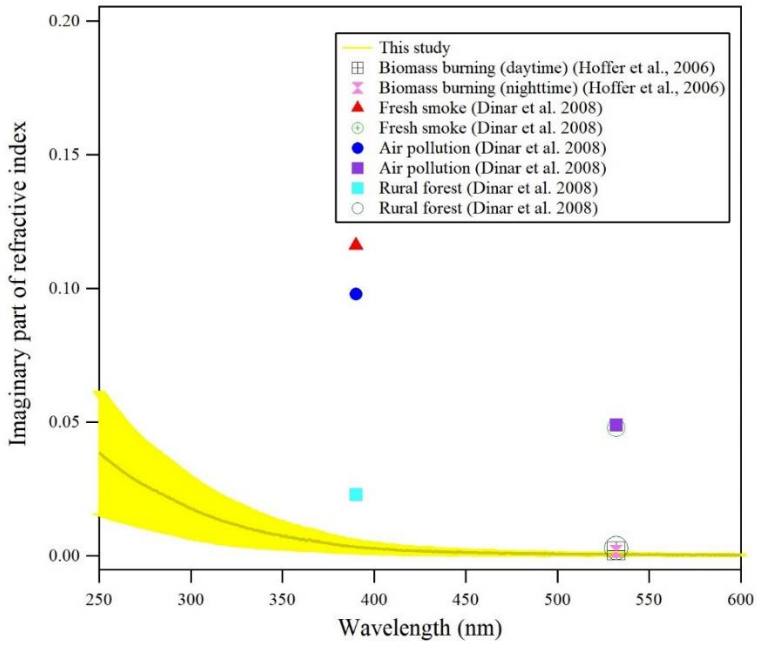

Figure S8. The imaginary part of the refractive index (*k*) for HULIS fractions. The solid line is the mean *k* values for HULIS of this study and the shaded area is the range within the standard deviation from the mean.

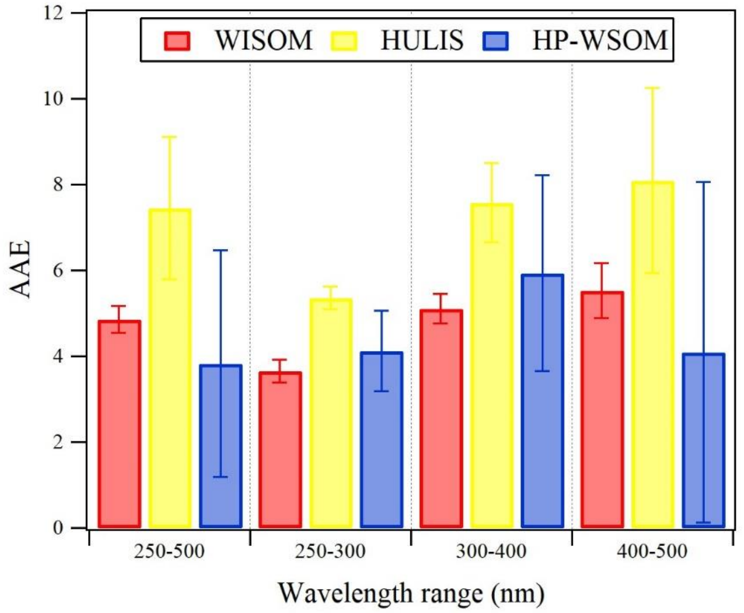

Figure S9. Absorption Ångström exponent (AAE) values at (a) 250-500 nm, (b) 250-300 nm, (c) 300-400 nm, and (d) 400-500 nm for WISOM, HULIS, and HP-WSOM.

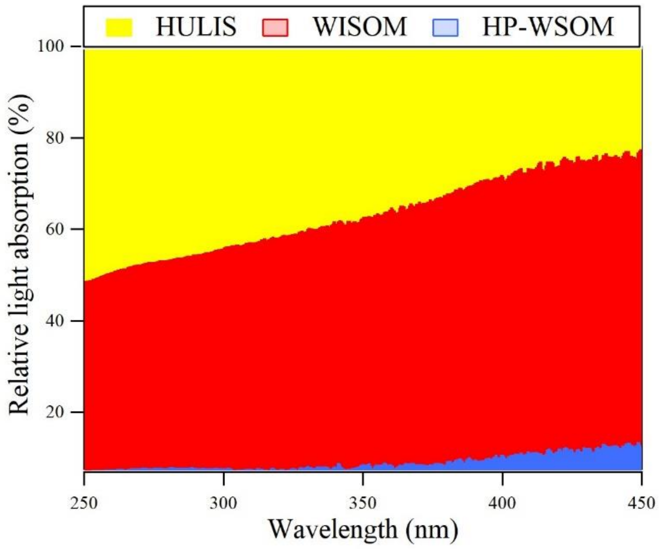

Figure S10. Stacked plot of the annual averages of the wavelength-resolved contributions of WISOM, HULIS, and HP-WSOM to the total light absorption by EOM.

Table S1. Sample information

Sample ID	Sampling start time (JST)	Sampling end time (JST)	Duration (h)	Sampled air volume (m^3)
TMK-A-009	0955, 22 June, 2012	1053, 29 June, 2012	168	11675.7
TMK-A-011	1107, 6 July, 2012	1034, 13 July, 2012	168	11479.6
TMK-A-013	1052, 20 July, 2012	1114, 31 July, 2012	264	18204.1
TMK-A-016	1300, 8 August, 2012	1150, 16 August, 2012	191	13058.5
TMK-A-018	1129, 24 August, 2012	1522, 31 August, 2012	168	11832.3
TMK-A-021	1335, 18 September 2012	1042, 26 September, 2012	189	12894.4
TMK-A-023	1115, 5 October, 2012	1029, 12 October, 2012	167	11505.4
TMK-A-025	1102, 23 October, 2012	1055, 2 November, 2012	240	16644.0
TMK-A-026	1120, 2 November, 2012	1224, 13 November, 2012	265	18458.7
TMK-A-029	1115, 20 December, 2012	1216, 28 December, 2012	193	13585.3
TMK-A-030	1150, 22 January, 2013	1115, 1 February, 2013	216	16514.5
TMK-A-033	1100, 20 February, 2013	1045, 27 February, 2013	168	11799.6
TMK-A-035	1147, 8 March, 2013	1131, 15 March, 2013	168	10856.3
TMK-A-038	1018, 3 April, 2013	1110, 12 April, 2013	217	15231.3
TMK-A-039	1000, 23 April, 2013	1054, 1 May, 2013	193	13381.6
TMK-A-041	1152, 8 May, 2013	1135, 15 May, 2013	168	11705.1
TMK-A-042	1200, 15 May, 2013	1045, 22 May, 2013	167	11662.2
TMK-A-044	1010, 29 May, 2013	1015, 4 June, 2013	144	10008.9

Table S2. Recoveries of samples using the SPE technique

Sample ID	Organic matter ($\mu g\ m^{-3}$)			SPE recovery (%)
	WSOM	HULIS	HP-WSOM	
TMK-A-009	1.89	1.46	0.34	94.78
TMK-A-011	1.17	0.75	0.57	109.56
TMK-A-013	0.90	0.72	0.25	106.86
TMK-A-016	1.77	1.55	0.26	103.00
TMK-A-018	1.15	0.89	0.29	102.22
TMK-A-021	1.23	1.02	0.22	101.19
TMK-A-023	0.86	0.71	0.22	107.43
TMK-A-025	1.40	0.97	0.37	95.59
TMK-A-026	0.54	0.47	0.10	105.05
TMK-A-029	0.79	0.50	0.37	110.84
TMK-A-030	0.47	0.34	0.10	94.82
TMK-A-033	0.99	0.77	0.25	102.25
TMK-A-035	0.77	0.61	0.21	106.65
TMK-A-038	1.23	0.82	0.41	99.58
TMK-A-039	1.63	1.27	0.43	103.75
TMK-A-041	0.84	0.49	0.48	110.89
TMK-A-042	1.14	0.73	0.56	108.07
TMK-A-044	0.67	0.48	0.21	101.16

Table S3. Concentrations (μg m^{-3}) of inorganic ions, EC, and OC in $PM_{0.95}$ over TOEF, Japan

Sample ID	Na^+	NH_4^+	K^+	NO_3^-	SO_4^{2-}	OC	EC
TMK-A-009	0.03	0.74	0.06	0.09	1.40	1.21	0.17
TMK-A-011	0.01	0.49	0.04	0.09	3.49	0.74	0.13
TMK-A-013	0.01	0.83	0.04	0.04	1.98	0.61	0.10
TMK-A-016	0.01	0.57	0.04	0.03	1.07	1.22	0.17
TMK-A-018	0.01	0.72	0.02	0.05	3.19	0.75	0.15
TMK-A-021	0.04	0.37	0.04	0.14	1.44	0.97	0.19
TMK-A-023	0.04	0.29	0.07	0.06	0.54	0.84	0.17
TMK-A-025	0.03	0.30	0.04	0.10	0.67	0.94	0.14
TMK-A-026	0.02	0.14	0.03	0.05	0.18	0.48	0.11
TMK-A-029	0.03	0.27	0.05	0.08	1.23	0.69	0.13
TMK-A-030	0.03	0.34	0.03	0.02	0.47	0.59	0.13
TMK-A-033	0.03	0.40	0.05	0.02	0.79	0.99	0.21
TMK-A-035	0.04	0.59	0.06	0.03	0.83	0.82	0.16
TMK-A-038	0.04	0.70	0.04	0.05	2.01	0.99	0.21
TMK-A-039	0.03	0.75	0.06	0.03	1.13	1.21	0.19
TMK-A-041	0.05	0.37	0.04	0.26	1.01	0.61	0.13
TMK-A-042	0.01	0.54	0.05	0.12	1.65	0.87	0.19
TMK-A-044	0.02	0.49	0.03	0.03	1.59	0.88	0.16
Mean ± SD	0.03 ± 0.01	0.49 ± 0.20	0.04 ± 0.01	0.07 ± 0.06	1.37 ± 0.88	0.86 ± 0.22	0.16 ± 0.03

Table S4. Seasonal and annual concentrations of OM and OC of HULIS, HP-WSOM, and WISOM[a]

	Organic matter (μg m^{-3})			Organic carbon (μg m^{-3})		
	HULIS	HP-WSOM	WISOM	HULIS	HP-WSOC	WISOC
All season	0.81 ± 0.34	0.31 ± 0.14	0.44 ± 0.11	0.36 ± 0.14	0.12 ± 0.05	0.29 ± 0.07
Summer (June-Aug.)	1.07 ± 0.40	0.35 ± 0.13	0.44 ± 0.12	0.48 ± 0.17	0.13 ± 0.05	0.30 ± 0.08
Autumn (Sept.-Nov.)	0.79 ± 0.25	0.23 ± 0.11	0.41 ± 0.07	0.36 ± 0.11	0.08 ± 0.04	0.28 ± 0.05
Winter (Dec.-Feb.)	0.54 ± 0.21	0.24 ± 0.14	0.39 ± 0.11	0.24 ± 0.09	0.09 ± 0.05	0.25 ± 0.06
Spring (Mar.- May)	0.73 ± 0.30	0.38 ± 0.14	0.48 ± 0.12	0.33 ± 0.12	0.14 ± 0.05	0.33 ± 0.09

[a] Mean ± SD

Table S5. Relative intensities (%) of seven fragment groups from the normalized HR-AMS spectra for each fraction in different seasons[a]

	C_xH_y	$C_xH_yO_1$	$C_xH_yO_{>1}$	$C_xH_yN_z$	$C_xH_yON_z$	$C_xH_yO_{>1}N_z$	CS
WISOM							
summer	76.12 ± 2.46	13.52 ± 1.31	7.12 ± 1.08	1.69 ± 0.39	1.37 ± 0.08	0.07 ± 0.03	0.10 ± 0.02
autumn	73.15 ± 2.89	16.22 ± 2.57	7.27 ± 1.06	1.86 ± 0.42	1.35 ± 0.15	0.06 ± 0.02	0.10 ± 0.01
winter	71.76 ± 3.70	14.96 ± 1.52	9.91 ± 1.66	1.58 ± 0.57	1.59 ± 0.48	0.08 ± 0.04	0.12 ± 0.04
spring	76.73 ± 2.81	12.82 ± 1.44	6.79 ± 1.44	2.14 ± 0.30	1.36 ± 0.25	0.05 ± 0.01	0.10 ± 0.05
HULIS							
summer	37.43 ± 1.09	37.59 ± 0.60	22.11 ± 1.68	1.73 ± 0.12	0.87 ± 0.16	0.14 ± 0.01	0.13 ± 0.01
autumn	39.92 ± 2.49	37.62 ± 0.83	18.93 ± 2.72	2.03 ± 0.24	1.20 ± 0.06	0.16 ± 0.02	0.14 ± 0.03
winter	38.24 ± 2.39	37.75 ± 0.79	20.40 ± 2.61	2.19 ± 0.07	1.09 ± 0.05	0.16 ± 0.02	0.16 ± 0.03
spring	38.32 ± 3.25	38.34 ± 0.70	19.40 ± 2.77	2.21 ± 0.11	1.39 ± 0.10	0.19 ± 0.03	0.15 ± 0.02
HP-WSOM							
summer	22.75 ± 1.63	38.53 ± 2.11	27.09 ± 2.07	8.80 ± 2.34	1.84 ± 0.37	0.11 ± 0.03	0.88 ± 0.21
autumn	24.35 ± 2.30	39.88 ± 2.82	26.33 ± 1.68	6.89 ± 1.16	1.72 ± 0.21	0.11 ± 0.04	0.56 ± 0.22
winter	24.14 ± 3.14	41.07 ± 1.98	27.54 ± 1.42	4.97 ± 0.62	1.62 ± 0.41	0.10 ± 0.02	0.56 ± 0.11
spring	23.21 ± 3.79	39.61 ± 2.95	28.33 ± 3.46	5.98 ± 1.63	1.83 ± 0.25	0.14 ± 0.03	0.90 ± 0.42

[a] Mean ± SD

Table S6. The mean ± SD of O/C, H/C, and OM/OC of organics in different fractions

Fractions	O/C	H/C	OM/OC
HULIS	0.80 ± 0.06	1.60 ± 0.07	2.22 ± 0.08
HP-WSOM	1.15 ± 0.10	1.49 ± 0.09	2.71 ± 0.11
WISOM	0.24 ± 0.05	1.89 ± 0.03	1.48 ± 0.06

Table S7. Seasonal and annual values (mean ± SD) of mass absorption efficiency (MAE) at 365 nm (m^2 g^{-1}) of EOM, WISOM, HULIS, and HP-WSOM.

	MAE at 365 nm			
	EOM	WISOM	HULIS	HP-WSOM
All season	0.21 ± 0.13	0.37 ± 0.22	0.14 ± 0.09	0.09 ± 0.07
Summer (Jun.-Aug.)	0.12 ± 0.02	0.23 ± 0.03	0.07 ± 0.02	0.10 ± 0.10
Autumn (Sep.-Nov.)	0.16 ± 0.04	0.28 ± 0.07	0.09 ± 0.03	0.09 ± 0.03
Winter (Dec.-Feb.)	0.47 ± 0.02	0.81 ± 0.14	0.31 ± 0.01	0.08 ± 0.03
Spring (Mar.- May)	0.19 ± 0.06	0.31 ± 0.10	0.15 ± 0.05	0.08 ± 0.07

Table S8. The annual values (mean ± SD) of the imaginary component of the refractive index (*k*) of EOM, HULIS, WISOM, and HP-WSOM

Wavelength (nm)	EOM	HULIS	WISOM	HP-WSOM
365	$6.14 \times 10^{-3} \pm 5.43 \times 10^{-3}$	$5.95 \times 10^{-3} \pm 3.86 \times 10^{-3}$	$1.09 \times 10^{-2} \pm 7.00 \times 10^{-3}$	$4.04 \times 10^{-3} \pm 3.22 \times 10^{-3}$
390	$4.26 \times 10^{-3} \pm 3.85 \times 10^{-3}$	$3.34 \times 10^{-3} \pm 2.40 \times 10^{-3}$	$8.13 \times 10^{-3} \pm 5.23 \times 10^{-3}$	$2.77 \times 10^{-3} \pm 2.84 \times 10^{-3}$
532	$1.01 \times 10^{-3} \pm 7.73 \times 10^{-4}$	$5.43 \times 10^{-4} \pm 5.21 \times 10^{-4}$	$2.01 \times 10^{-3} \pm 9.65 \times 10^{-4}$	$1.26 \times 10^{-3} \pm 1.53 \times 10^{-3}$

Table S9. Seasonal and annual values (mean ± SD) of E_2/E_3 of HULIS, HP-WSOM, and WISOM.

	WISOM	HULIS	HP-WSOM
All season	5.13 ± 0.30	10.07 ± 1.95	8.55 ± 5.17
Summer (Jun.-Aug.)	5.30 ± 0.48	10.97 ± 2.43	6.57 ± 3.00
Autumn (Sep.-Nov.)	5.05 ± 0.22	11.09 ± 1.20	8.81 ± 6.71
Winter (Dec.-Feb.)	4.90 ± 0.03	9.13 ± 0.75	8.96 ± 3.96
Spring (Mar.- May)	5.16 ± 0.16	9.12 ± 1.96	9.81 ± 6.65

Table S10. Annual values of the AAE and parameter *a* (mean ± SD) of aerosol (EOM + EC), EOM, HULIS, HP-WSOM, and WISOM[1]

Wavelength (nm)	Aerosol (EOM+EC)	HULIS	WISOM	HP-WSOM
250-500	1.18 ± 0.10 ($2.43 \times 10^{4} \pm 1.97 \times 10^{4}$)	7.46 ± 1.66 ($3.16 \times 10^{30} \pm 1.34 \times 10^{31}$)	4.86 ± 0.31 ($2.06 \times 10^{13} \pm 4.11 \times 10^{13}$)	3.83 ± 2.64 ($1.77 \times 10^{21} \pm 7.50 \times 10^{21}$)
250-300	1.31 ± 0.14 ($6.22 \times 10^{4} \pm 7.71 \times 10^{4}$)	5.36 ± 0.26 ($2.02 \times 10^{14} \pm 3.71 \times 10^{14}$)	3.66 ± 0.27 ($1.74 \times 10^{10} \pm 3.02 \times 10^{10}$)	4.12 ± 0.94 ($4.65 \times 10^{13} \pm 1.86 \times 10^{14}$)
300-400	1.19 ± 0.11 ($2.74 \times 10^{4} \pm 2.76 \times 10^{4}$)	7.58 ± 0.92 ($1.77 \times 10^{22} \pm 7.10 \times 10^{22}$)	5.11 ± 0.35 ($2.31 \times 10^{14} \pm 7.42 \times 10^{14}$)	5.94 ± 2.28 ($2.00 \times 10^{25} \pm 8.48 \times 10^{25}$)
400-500	1.06 ± 0.04 ($9.71 \times 10^{3} \pm 2.91 \times 10^{3}$)	8.10 ± 2.15 ($9.63 \times 10^{61} \pm 4.09 \times 10^{62}$)	5.53 ± 0.64 ($1.73 \times 10^{16} \pm 3.76 \times 10^{16}$)	4.09 ± 3.97 ($3.79 \times 10^{32} \pm 1.61 \times 10^{33}$)

[1]AAE and *a* were derived by the fitting of the measured light absorption spectra by Equation 3

REFERENCES

1. Varga, B.; Kiss, G.; Ganszky, I.; Gelencsér, A.; Krivácsy, Z. Isolation of water-soluble organic matter from atmospheric aerosol. Talanta 2001, 55 (3), 561-572.
